# A Review of Toxins from Cnidaria

**DOI:** 10.3390/md18100507

**Published:** 2020-10-06

**Authors:** Isabella D’Ambra, Chiara Lauritano

**Affiliations:** 1 Integrative Marine Ecology Department, Stazione Zoologica Anton Dohrn, Villa Comunale, 80121 Napoli, Italy; 2 Marine Biotechnology Department, Stazione Zoologica Anton Dohrn, Villa Comunale, 80121 Napoli, Italy; chiara.lauritano@szn.it

**Keywords:** venom, phospholipase, metalloproteinases, ion channels, transcriptomics, proteomics, biotechnological applications

## Abstract

Cnidarians have been known since ancient times for the painful stings they induce to humans. The effects of the stings range from skin irritation to cardiotoxicity and can result in death of human beings. The noxious effects of cnidarian venoms have stimulated the definition of their composition and their activity. Despite this interest, only a limited number of compounds extracted from cnidarian venoms have been identified and defined in detail. Venoms extracted from Anthozoa are likely the most studied, while venoms from Cubozoa attract research interests due to their lethal effects on humans. The investigation of cnidarian venoms has benefited in very recent times by the application of omics approaches. In this review, we propose an updated synopsis of the toxins identified in the venoms of the main classes of Cnidaria (Hydrozoa, Scyphozoa, Cubozoa, Staurozoa and Anthozoa). We have attempted to consider most of the available information, including a summary of the most recent results from omics and biotechnological studies, with the aim to define the state of the art in the field and provide a background for future research.

## 1. Introduction to the Phylum Cnidaria

**Figure 1:**
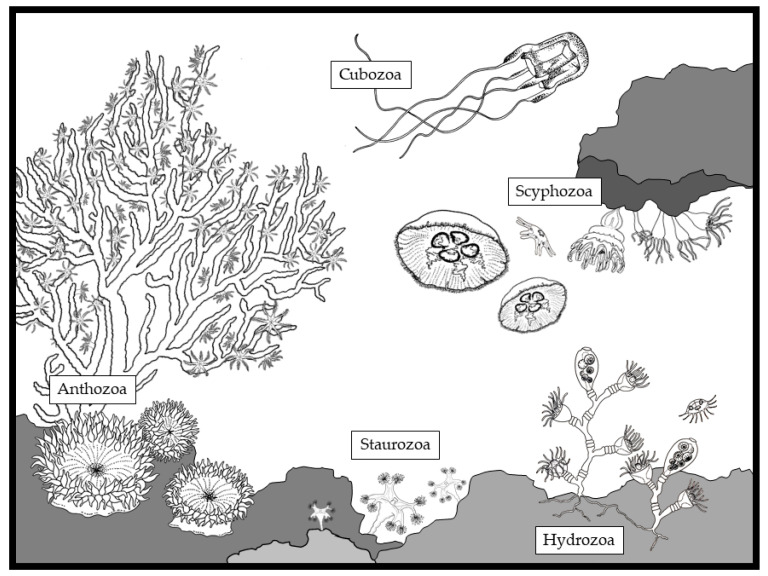
The diverse organisms belonging to the phylum Cnidaria within the main classes: Hydrozoa, Scyphozoa, Cubozoa, Staurozoa and Anthozoa (drawing by Louise Merquiol).

The phylum Cnidaria includes five main classes: Hydrozoa, Scyphozoa, Cubozoa, Staurozoa and Anthozoa (Table 1; Figure 1). Basically, the unit organism in Cnidaria is the polyp, a sessile, small (from few millimeters to less than two centimeters) gastrovascular cavity surrounded by three layers (an internal endodermis, and an external ectodermis with an intermediate matrix, the mesoglea). The mouth of the polyp is surrounded by tentacles, which facilitate prey capture [1]. Polyps form different types of colonies in Hydrozoa and Anthozoa. Conversely, they live as single individuals in Scyphozoa, Cubozoa and Staurozoa. In addition to the polyp stage, Hydrozoa, Scyphozoa, Cubozoa and Staurozoa have a freely swimming pelagic stage (medusa), which co-exists with the polyp stage. The fact that Hydrozoa, Scyphozoa and Cubozoa have a metagenic life cycle (Table 1) suggests that they may have evolutionary separated from Anthozoa, which have only the polyp stage (Table 1). Phylogenetic analyses confirmed that Anthozoa appeared earlier than the other three classes within the evolutionary history of Metazoa, because they possess a circular DNA, in contrast with Hydrozoa, Scyphozoa and Cubozoa, which have a linear DNA [2].

Both pelagic and benthic organisms belonging to this phylum possess complex, specialized organelles (cnidae) made of an amino acid matrix secreted by the Golgi system. The structure of the cnidae is common across Cnidaria: a capsule with collagen walls filled with a venom with a coiled hollow thread-like tubule. The tubule everts after a mechanical stimulation. Most tubules, particularly in the nematocysts, are able to penetrate the skin and inject the venom contained in the capsule [3]. The threads have an enlargement at their basal portion (the shaft) and bear numerous and different spines. The classification of cnidae is based on the shape of the shaft and the type and distribution of the spines [3,4]. The main classification includes nematocysts, spirocysts and ptychocytes [3,4,5]. Nematocysts have two walls surrounding the capsule. Conversely, the capsule of spirocysts has a single wall that is very thin. Hydrozoa, Scyphozoa, Cubozoa and Staurozoa possess various types of nematocysts, while spirocytes have been found in Zoantharia and ptychocytes appear to be a characteristic of Ceriantharia tubes (Table 1) [3,4,5]. Ames et al. [7] described “cassiosomes” in the upside-down scyphomedusa *Cassiopea xamachana* using a combination of histology, microscopy, microfluidics, videography, molecular biology and mass spectrometry-based proteomics. Cassiosomes are complex stinging-cell structures found in *C. xamachana* mucus and likely used to kill prey. Cassiosomes are made up by an outer epithelial layer of nematocytes that is filled in the internal part by endosymbiotic dinoflagellates, which are known to be hosted within the mesoglea of the scyphomedusa.

The mechanism of nematocyst discharge is activated by chemical and physical stimulation. When the cnidocil is solicited, the thread, which is enveloped inside the capsule, is pushed by the osmotic pressure outside the cell, and eventually penetrates the object, which stimulated the cnidocil [1]. In most organisms, the injection induces paralysis, which facilitates the following capture of the organism. The prey capture mechanism described above is basically the same for all Cnidaria, polyp and medusa stages alike [8]. However, as reported by Moran and co-workers [9], Cnidaria toxins may be localized in both nematocysts and ectodermal gland cells, depending on the species. For example, Nv1 neurotoxin from the sea anemone *Nematostella vectensis* is confined to the ectodermal gland cells, while *Anemonia viridis* Type I toxins are localized in both nematocysts and ectodermal gland cells [9].

## 2. Venoms of Cnidaria

The composition of cnidarian venoms is not known in detail, but they appear to contain mainly a variety of proteinaceous (peptides, proteins, enzymes and proteinase inhibitors) together with non-proteinaceous compounds (purines, quaternary ammonium compounds, biogenic amines and betaines) [10,11,12,13,14] (Table 2). The main venom components identified to date have a maximum molecular mass of 220 kDa. Phopholipase A_2_ appears to be common across all cnidarian classes [15]. Anthozoans contain several inhibitors of both sodium and potassium voltage-gated channels [11]. The channel binding site is not always known. However, as reported by [11,16], receptor site three has been identified as the binding site for sea anemone Type I and Type III sodium channel inhibitors. Toxins have been recently reviewed in Scyphozoa [13,14,17] and in sea anemones (Actinaria) [11,14]. In this review, we provide an updated synopsis of the main compounds identified to present in the venom of cnidarians and discuss the recent application of omics and biotechnological tools in the field.

### 2.1. Phospholipase A_2_

Phospholipase A_2_ induces the breakdown of glycerophospholipds, which produces lysophospholipid and fatty acids, such as the arachidonic acid [162]. The metabolites derived from arachidonic acid (prostaglandins, thromboxanes and leukotrienes) control a variety of cellular functions, including dietary lipid catabolism, in cell membrane metabolism and inflammatory diseases [163].

Phospholipase A_2_ is common in mammalians but also across venomous animals. It has been identified in reptiles (snakes and anguimorph lizard), centipedes, insects (their bristles, proboscises, and stingers), arachnids (scorpions, spiders, and ticks), cnidarians and cephalopods [15,164]. Toxic functions of phospholipase A_2_ in cnidarian venoms have been proposed to include defense, immobilization and digestion of prey [15]. The first cnidarians phospholipase A_2_ fully sequenced was published in 2002 for *Adamsia carcinoapados* [82].

### 2.2. Metalloproteinases

Metalloproteinases include a large variety of proteinase enzymes, which host a metal atom to perform their catalytic activity. They are found in the venom of terrestrial animals, such as centipedes, snakes and ticks [164,165]. They induce hemorrhage and necrosis by degrading the extracellular matrix and preventing blood clot formation [164,166]. These functions are commonly associated with several symptoms following a sting (skin damage, edemas, blister formation, myonecrosis and inflammation) [166]. They appear to be common in stinging jellyfish, such as *Stomolophus meleagris* [59] and *Chironex fleckeri* [66].

### 2.3. Voltage-Gated Sodium and Potassium Channel Toxins

Any ion channel family generally comprises various subtypes with particular physiological, pharmacological and structural characteristics [167]. The voltage-gated ion channels play a crucial role in the excitability of cells and neuromuscular transmission of signals. Voltage-gated ion channels activate non-selective pores within membranes by which the ions can pass using the electrochemical gradient across the membrane itself [167]. When this mechanism is altered, the transmission of signals through the neurons and muscles is critically changed too, which can lead to certain disorders, including paralysis.

Voltage-gated sodium channel toxins have been isolated since the 1970s in Anthozoa [11], and they account for the most abundant fraction in their venom [16]. These toxins, whose molecular mass ranges from 3.5 to 6.5 kDa, are able to bind specifically with the receptor site three of the sodium channel, and regulate their functioning [168]. By controlling the opening and closing of the sodium channel, the toxins control the electrical signals that encode and propagate vital information across long distances. The activity of the sodium channel toxins suggests that they may find application as pain blockers. Conversely, Kalima and co-workers [169] suggested that the sodium channel inhibitors extracted from *Heteractis crispa* were not appropriate for pharmacological applications, but might be used to study the mechanisms beyond sodium transportation and to produce insecticides.

Compared to the voltage-gated sodium channel toxins, voltage-gated potassium channel toxins were discovered during the 1990s [11]. Recently, toxins acting on the voltage-gated potassium channel have been investigated for the treatment of multiple sclerosis and other autoimmune diseases [170].

### 2.4. Kunitz-Type Proteinase Inhibitors

Kunitz-type proteinase inhibitors have been identified in several diverse species of cnidarians, but exclusively anthozoans (Table 2). Their active domains, also known as Kunitz/BPTI domains, are relatively small, with a molecular weight of about 6 kDa. Before the discovery of potassium channel Type II toxins, sea anemone proteinase inhibitors were considered to serve as inhibitors of endogenous proteinases in the sea anemones themselves and as protectors of the toxins injected into prey and predators from rapid degradation. However, the identification of potassium channel toxins with proteinase inhibitory activity suggests that the offensive role of sea anemone proteinase inhibitors, by paralyzing prey, may be more important than their defensive role [171]. The Kunitz-type IQ-peptide HMIQ3c1, extracted from the venom of *Heteractis magnifica*, has shown neuroprotective activity, which may find application in the treatment of neurodegenerative diseases, such as Alzheimer’s [138]. Another Kunitz-type proteinase inhibitor that may support the treatment of Parkinson disease is HCIQ2c1 extracted from *Heteractis crispa* [121]. This study shows for the first time that Kunitz-peptides significantly increase neuroblastoma cell viability in an in vitro 6- hydroxydopamine-induced neurotoxicity model of Parkinson’s disease [121].

### 2.5. TRPV1 Channel Inhibitors

The transient receptor potential vanilloid 1 (TRPV1s) are transmembrane non-selective cation channels in mammalian peripheral and central neuronal systems. Because they control the response of the neurons during inflammation, they are considered among the most important molecular triggers of pain stimuli [172]. The first TRPV1 peptide inhibitor from sea anemone venoms was τ-SHTX-Hcr2b (APHC1) from *Heteractis crispa* [127]. Research on the TRPV1 inhibitors has continued on this species, from which the other three homologous peptides, τ-SHTX-Hcr2c (APHC2), τ-SHTX-Hcr2d (APHC3) and HCRG21, have been isolated [126,129]. These three peptides showed analgesic activity in an in vivo heat stimulation model [130,139]

### 2.6. TRPA1 Channel Modulators

The transient receptor potential ankyrin 1 ion channel (TRPA1) is a nociceptor [173] that has been suggested to serve as the main mechanical and chemical stress sensor. Agonists of TRPA1 activate the sensory neurons in vivo, which cause acute pain, thermal and mechanical hyperalgesia and neurogenic inflammation. This receptor is similar to the transient receptor potential vanilloid 1 (TRPV1), with which it is usually co-expressed [174]. Another TRPA1 modulator, named Ms 9a-1, was isolated from *Metridium senile* and showed an analgesic effect [141]. In addition, Logashina and co-workers [159] isolated Ueq 12-1 from *Urticina eques*, which belongs to a group of toxins never identified previously. The antibacterial activity and the moderate enhancement of TRPA1 suggest that this peptide may find large application as an analgesic with antibacterial properties [159].

### 2.7. ASICs Channels Modulators

ASICs are sodium-selective, acid-sensing ion channels in the peripheral nervous system, particularly activated during inflammation and ischemia. Peptides π-AnmTX Ugr 9a-1 from the venom of the sea anemone *Urticina grebelnyi* [160] and PhcrTx1 from *Phymanthus crucifer* [149] target ASIC channels. These peptides are cross-linked by two disulfide bridges and have no sequence homology to other sea anemone neurotoxin peptides [10]. In addition, Hcr 1b-1, Hcr 1b-2, Hcr 1b-3 and Hcr 1b-4 have been also identified from the sea anemone *Heteractis crispa* and have shown neurotoxic activity by inhibiting the ASIC channels. In particular, Hcr 1b-1 is a specific ASIC3 inhibitor and Hcr 1b-2 is able to inhibit both ASIC1a and ASIC3 channels; the others are specific for the ASIC1a channel [123].

### 2.8. Beta-Defensin Like Alpha-Amylase Inhibitors

Among animals, amylase inhibitors have been identified only in sea anemones, which are among the most ancient phyla that appeared on Earth [2]. Helianthamide, a potent inhibitor of human pancreatic α-amylase produced by the Caribbean Sea anemone *Stichodactyla helianthus*, is the first representative of a new group of amylase inhibitors that was isolated in 2016 [175], while Magnificamide is the second representative of such inhibitors. Both peptides appear to be promising compounds to manage obesity and type 2 diabetes [139]. The biological relevance of the presence of α-amylase inhibitors in the Cnidaria venoms remains largely unexplained. It is hypothesized that inhibition of the α-amylase activity intervenes with the metabolism of carbohydrates, a major source of energy for many organisms [139].

## 3. Omics Applied to Cnidaria to Identify Chemical Defences

Recently, omics technologies (genomics, transcriptomics, proteomics and metabolomics) have helped to better understand the ecology, distribution and defence strategies of marine organisms, as well as having speeded up studies on their possible biotechnological applications. Available studies on defence strategies in Cnidaria mainly involve deadly toxins released to capture prey, defend themselves or fight for territorial acquisition.

Considering the available genomes, there are 6 Hydrozoa, 10 Scyphozoa, 4 Cubozoa, 18 Anthozoa, 1 Staurozoa and 7 Myxozoa genomes deposited in the public database GenBank (Table 3). For many of them, sequences have been deposited during the last year and the related publications are not available yet. Several transcriptomic, proteomic and metabolomic studies are available as well, but few of them focused on defence activities. Recently, in order to shed light on the molecular basis of coral responses to environmental changes and reef-building coral strategies for conservation, several genomes and transcriptomes have been sequenced (Table 3).

Gene families encoding toxins are found in many venomous species, but there is still limited understanding of their evolution. Genome sequencing and analyses have started to help shed light on toxins and their synthesis. Sea anemones may produce various toxic compounds (such as peptide toxins found in their venoms), which can have potential therapeutical applications. For example, the genome of the sea anemone *Nematostella vectensis* enabled to study a gene family whose neurotoxin product, Nv1, affects voltage-gated sodium channels [142]. In particular, the gene family members identified (116.25.1, 116.27.1, 116.28.1, 116.37.1, 116.39.1, 116.40.1, 116.41.1 and 116.45.1) clustered in a highly repetitive approximately 30-kb genomic region and encoded the toxin Nv1. Transcriptome analyses of the sea anemone *Nematostella vectensis* allowed to identify the sequences encoding precursor proteins homologous to the Nv1 toxin (named as Nv4, Nv5, Nv6, Nv7 and Nv8). In addition, Sachkova and co-workers showed that the new toxins, Nv4 and Nv5, were lethal for zebrafish larvae but harmless to arthropods, and were localized to ectodermal gland cells in larvae [143]. Recently, the genome of *Actinia tenebrosa* was sequenced and annotated. Bioinformatic analyses showed that the genes encoding toxins contributed to a significant proportion of the lineage-specific genes and gene families, giving new insights into the evolution of toxins and possibly guiding the discovery of novel bioactive compounds from Cnidaria.

Regarding the transcriptome data, there are 35 BioProjects and 62 sequence read archives (SRA) deposited in GenBank (accessed on August 25th, 2020). A limited number of them are transcriptomes related to defence strategies in Cnidaria. An example of a transcriptomic study (using an Illumina HiSeq 2500 automatic sequencing platform) was performed by Huang et al. [193] for the sea anemone *Protopalythoa variabilis*. The transcriptome analyses identified various predicted polypeptides with canonical venom protein features belonging to various toxin families: neurotoxic peptides, hemostatic and hemorrhagic toxins, pore-forming proteins, proteinase inhibitors, mixed-function venom enzymes and venom auxiliary proteins. Two of these predicted toxin products, ShK/Aurelin-like peptide and a novel anthozoan neurotoxin-like peptide, were further investigated and displayed potent in vivo neurotoxicity that impaired swimming in larval zebrafish. A complex array of venom-related transcripts was identified and some of them are reported in Cnidaria for the first time, giving new insights in toxin distribution among species and their evolution.

Another transcriptome study (using an Illumina HiSeq 2500 Sequencing System) by Ames et al. [194] focused on sequencing and analysing the transcriptomes of adult and larval tissues of the cubozoan *Alatina alata*, which is emerging as a cnidarian model because it forms predictable monthly nearshore breeding aggregations in tropical to subtropical waters. Differential expression analyses were performed to identify the candidate genes involved in nematogenesis and venom production, giving a boost for further investigations on the evolution of distinctive characteristics of cubozoans. In particular, various candidate genes implicated in predation, defence, vision and phototransduction pathways, sexual reproduction and embryogenesis were identified, which may be considered for further studies on cubozoan/cnidarian evolution.

Li and collaborators [59] used a combination of transcriptomic and proteomic approaches in order to shed light on the toxic jellyfish *Stomolophus meleagris*. Venom proteomics was performed by tryptic digestion of the crude venom followed by RP-HPLC separation and MS/MS analysis of the tryptic peptides. The venom gland transcriptome was analysed using an Illumina sequencing platform (HiSeq™ 2000) with de novo assembly. A total of 218 toxins were identified, including the C-type lectin, phospholipase A_2_, potassium channel inhibitors, serine proteinase inhibitors, metalloproteinases and hemolysins [59].

Very recently, Koch and Grimmelikhuijzen [195] used a software to annotate neuropeptides in the publicly available genomes and transcriptomes from Cubozoa, Scyphozoa and Staurozoa (which all belong to the subphylum Medusozoa) and compared these results with the neuropeptides present in Octocorallia (class Anthozoa). Three to six neuropeptide preprohormone genes were identified within members of the abovementioned cnidarian classes, each coding for several (up to thirty-two) similar or identical neuropeptide copies [195]. Two of these neuropeptide preprohormone genes were present in all the cnidarian classes investigated, and they were supposed to be among the first neuropeptide genes evolved in cnidarians.

Ponce et al. [196] used an integrated transcriptomic (using an Illumina HiSeq 2000 system) and proteomic approach to identify putative toxins and their potential role in the venom of the scyphozoan *Chrysaora fuscescens*. The de novo tentacle transcriptome contained more than 23,000 contigs and a total of 163 proteins were identified in the venom proteome of *C. fuscescens*. Of these proteins, 27 were classified as putative toxins and grouped into six protein families: proteinases, venom allergens, C-type lectins, pore-forming toxins, glycoside hydrolases and enzyme inhibitors. Interestingly, other putative toxins were identified in the transcriptome, but not in the proteome (they were probably not expressed in the moment of the experiment), such as other proteinases, lipases and deoxyribonucleases. Sequence analysis also revealed the presence of ShKT domains (domains found in a group of potent potassium channels blockers originally isolated from sea anemones) in two putative venom proteins from the proteome and 15 from the transcriptome, suggesting potential ion channel blockade activities. Analysis of the venom proteomes has recently become more feasible and is generally used to study which are the possible proteins responsible for the most severe symptoms of jellyfish stings and also for guiding the identification of proteins with potential therapeutic applications [196].

Knowledge of toxic peptides in cnidarians is very limited due to the small number of toxins (mainly from sea anemones) identified to date by traditional protein analyses. Protein analyses of nematocysts of the hydromedusa *Olindias sambaquiensis* allowed to identify 29 putative toxins using a high throughput proteomics platform [24]. The data revealed 29 potential toxins homologous to toxic proteins from diverse animal phyla, including cone snails, snakes, spiders, scorpions, wasps, bees, parasitic worms and other Cnidaria. The presence of several toxic enzymes has been observed, such as sphingomyelin phosphodiesterase B (previously described in some spider venoms) and a prepro-haystatin P-IIId snake venom metalloproteinase, which is very rare, even within snake venoms.

Similarly, Brinkman and co-workers [197] used a proteomic approach to identify the protein components of the venom from the cubozoan *Chironex fleckeri*. Collectively, 61 proteins were identified, including toxins and proteins important for nematocyte development and nematogenesis. Venom proteins and their post-translational modifications were also further characterized using toxin-specific antibodies and phosphoprotein/glycoprotein-specific stains. Data showed that glycosylation is a common post-translational modification of the toxin family. In addition, a lack of cross-reactivity by toxin-specific antibodies was observed, suggesting that there is significant divergence in toxin structures.

A proteomic analysis of the most venomous jellyfish in the Mediterranean Sea, *Pelagia noctiluca*, was performed to study the jellyfish proteins involved in defence, body constituents and metabolism, and also explored the potential application of such bioactive molecules [198]. The results allowed to identify for the first time in *P. noctiluca* a zinc metalloproteinase, a red fluorescent protein (RFP) and a peroxiredoxin. Zinc metalloproteinase was previously reported in the venom of other jellyfish species, it has a ShK toxin domain and therefore should be implicated in *P. noctiluca* toxicity. The RFP is an important family of proteins with possible applications as molecular markers, while peroxiredoxin is a known antioxidant and suggest this scyphozoan species as a potential natural source of antioxidants and anti-UV radiation agents.

Zaharenko et al. [105] reported the first peptide mass fingerprint of the sea anemone *Bunodosoma cangicum* venom and, in particular, of the neurotoxic (on crabs) fraction named FrIII. This proteomic approach allowed them to identify some novel peptides. FrIII was purified obtaining 41 fractions. Between the 81 components present in these fractions, three groups of toxic peptides were identified. Bcg 25.96, Bcg 28.19 and Bcg 30.24 of about 4 kDa were identified as Type I potassium channel toxins. Bcg 31.16, Bcg 28.78, Bcg 25.52 and Bcg 29.21, with a molecular mass between 4 and 5 kDa, showed sequence features characteristic of toxins that may target different types of ion channels (e.g., the human cardiac potassium channel HERG and the human acid-sensing ion channel ASIC3). Finally, Bcg 21.75, Bcg 23.41 and Bcg 21.00 were characterized as a possible new class of potassium channel blockers neurotoxic to crabs.

Comparative proteomics (by mass spectrometry analyses) was performed to compare the soluble nematocyst’s proteome from the sea anemone *Anemonia viridis*, the jellyfish *Aurelia aurita* and the hydrozoan *Hydra magnipapillata* [199]. Even if several protein domains were shared between the three organisms’ nematocyst content, suggesting common proteome functionalities, only six proteins were identified as shared by the three organisms. The authors suggested that conserved proteins among distantly related cnidarians are likely to be important for nematocyst structure or function. The six common proteins were nematogalectin (structural component of the nematocyst tubule), elongation factor-1α (involved in translation), dickkopf (a Wnt ligand, multipurpose protein that is suggested to also serve as a toxin), the chaperone protein heat shock protein 70 or HSP70 (which promotes the correct protein folding) and two proteins with unknown function. The venoms of *Hydra magnipapillata* and *Aurelia aurita* appeared more similar to each other, composed mainly of cytotoxins and enzymes, while the venom of *Anemonia viridis* was characterized by peptide neurotoxins. Altogether, the results suggested that the protein pools were unique to each organism and potentially to each nematocyst type.

Comparative proteomics was also used to determine the venom composition of the scyphozoan *Chrysaora lacteal,* the two cubozoans *Tamoya haplonema* and *Chiropsalmus quadrumanus,* and the other 5 cnidarian venom proteomes available in literature (the anthozoans *Anemonia viridis* and *Acropora digitifera*; the hydrozoans *Olindias sambaquiensis* and *Hydra magnipapillata*; and the scyphozoan *Aurelia aurita*) [200]. The comparative analysis allowed to identify 28 putative toxin protein families, many on them identified for the first time in Cnidaria (e.g., a glycosyl hydrolase 56, a lipase, huwentoxin-1 and latarcin).

Metabolomic studies are very scarce. Metabolomic profiling of three genetically different individuals of the same coral species, the threatened coral *Acropora cervicornis* [201], was performed. The data showed differences in protein synthesis among the genotypes, suggesting that different genotypes may have different abilities, related to growth and stress tolerance, to persist under present and future environmental conditions. Research in this field will have the potential to support a guided selection of robust genotypes for restoration programs.

## 4. Cnidaria Bioprospecting

The crude venom extracted from cnidarians has a wide range of effects on humans, such as dermonecrosis, edema, diffused neurotoxicity, motorial and respiratory problems, cardiovascular symptoms, hypotension and occasionally death [17]. Cytotoxic, cytolytic, hemolytic and neurotoxic activities are the most common effects observed for crude Cnidaria venom (Table 2). Cnidaria compounds and toxins have attracted attention, not only for their effects on humans, but also because they can be very useful molecular probes for the study and analysis of ion channels involved in electrical signaling and immune responses, which can have biomedical interest [202]. In 1913, Charles Robert Richet won the Nobel Prize in Physiology or Medicine for his work on anaphylaxis, a potentially life-threatening immune hypersensitivity reaction to an antigen, discovered during experiments with a sea anemone (*Actinia*) toxin [202].

As reported in the previous paragraphs, Cnidaria venoms may contain enzymes, pore-forming toxins, neurotoxins and enzyme inhibitors. Neuroactive peptides targeting the central nervous system, due to their affinity with sodium and potassium channels, may provide new lead compounds to treat neurological diseases caused by ion channel dysfunctions (e.g., neurodegenerative diseases, epilepsy, as well as acute and chronic pain) [170]. Only one peptide from Cnidaria, named ShK-186 or dalazatide, is currently in clinical trials for the treatment of autoimmune diseases. In particular, dalazatide is a potassium channel (Kv1.3) blocker and has successfully completed Phase 1 within clinical trials and is about to enter Phase 2 of the trials for the treatment of multiple sclerosis and rheumatoid arthritis [203].

Venom components have been shown to possess interesting bioactivities for possible pharmaceutical applications [14,204]. As reviewed by Mariottini and Pane [14], several compounds/extracts from Cnidaria have shown cytotoxic and cytolytic activities on various mouse and human cell lines. Studies also tested cnidarian compounds on normal cells in order to show their selectivity. Compounds/extracts from Cnidaria have shown to have activity against leukemia, colon carcinoma, lymphoma, lung adenocarcinoma, glioblastoma, melanoma, ovarian adenocarcinoma, breast adenocarcinoma, cervix carcinoma, epidermoid carcinoma, glioblastoma, hepatoma, pheochromocytoma, prostate and pancreatic carcinoma, fibrosarcoma, renal and central nervous system cancer. The lowest IC_50_ of 0.000005 µg/mL was observed for sesquiterpenes isolated from *Isis hippuris* (Anthozoa, Octocorallia) and active on human colon adenocarcinoma HT-29 and mouse lymphoma P388. Five new polyoxygenated marine steroids, named punicinols A, B, C, D and E, were isolated from the gorgonian *Leptogorgia punicea* (Anthozoa, Octocorallia) and showed in vitro cytotoxic activity (evaluated by using the sulforhodamine B assay) against human lung cancer A549 cells [205]. Punicinols A and B were the most active, with IC_50_ values of 9.7 μM and 9.6 μM, respectively. These two compounds were also tested in combination with paclitaxel, a well-known cytotoxic compound, and showed synergistic effects. Cell-cycle analysis showed that punicinol A induced the arrest of the Sub G0/G1, while punicinol B the G2M phase. Punicinols A and B also showed effects on the clonogenic potential of A549 cells, completely inhibiting the growth of cancer cells after 24 h of treatment and 10 days for cell proliferation monitoring. Further studies (e.g., by also using normal cells) can clarify punicinols A and B activity and may propose them combined with paclitaxel in tumor chemotherapy.

*Pelagia noctiluca* (Scyphozoa) venom constituents have shown anticancer and anti-inflammatory activities [206]. *P. noctiluca* venom was fractioned using Sephadex G75; four fractions were obtained and their anti-proliferative activity was tested on three cancer cell lines (human bladder carcinoma RT112, glioblastoma U87 and human myelogenous leukemia K562) and on human peripheral blood mononuclear cells (PBMC) obtained from the blood of healthy volunteers. Three fractions (F1, F2 and F3) exhibited cytotoxic activity, in a dose-dependent manner, but had little effect on PBMC, showing selective anti-proliferative activity. *P. noctiluca* also showed a dose-dependent anti-inflammatory activity, as the nitric oxide (NO) production inhibition activities in IFN-ɣ/LPS stimulated murine RAW 264.7 macrophages. F1 was the most active, inducing an 84% decrease of NO production. In addition, molecular analyses showed that F1, F2 and F3 significantly and dose-dependently inhibited the inducible nitric oxide synthase (iNOS) mRNA expression, showing fraction activity at the transcriptional level. F4 had no effects.

Considering the increasing human consumption of sea anemones as food, Silva et al. [207] evaluated the effects of aqueous extracts, in order to mimic the route of ingestion, of two species of sea anemones, *Actinia equina* and *Anemonia sulcata*. They found that the major compound present in the extracts was the methylpyridinium alkaloid homarine and, considering that it was not commercially available, also synthetized it. They first tested the cytotoxic effects of the two extracts in murine RAW 264.7 macrophages. At 24 h, the aqueous extract of *A. equina* showed the highest toxicity and a concentration-dependent toxic effect (IC_50_ = 0.629 mg/mL). At higher incubation periods, the *A. equina* extract did not show major differences, while the extract of *A. sulcata* was more toxic with time, causing a reduction in cell viability around 62.63% ± 7.01% after 72 h at the highest concentration tested (1 mg/mL). The synthetized homarine displayed higher cytotoxicity at 48 h (decreasing cell viability by 41.93% ± 7.35% at 1 mg/mL). In addition, analyses showed that incubation of cells with *A. sulcata* (1 mg/mL) and *A. equina* (0.5 mg/mL) extracts resulted in increased activity of caspase-3, suggesting a caspase-dependent cell death in macrophages. In vitro anti-inflammatory activity of the two extracts and homarine was also evaluated in a murine macrophage model of inflammation, using a lipopolysaccharide (LPS)-induced RAW264.7 cell line. Results showed that they were able to reduce the LPS-induced levels of NO (extracts of *A. sulcata* at 0.374 mg/mL, *A. equina* at 0.125 mg/mL and homarine at 0.25 mg/mL) and intracellular reactive oxygen species (ROS) (the two extracts were able to decrease ROS, while homarine could not) in macrophages and that both extracts and homarine were able to inhibit phospholipase A_2_, a pivotal enzyme in the initial steps of the inflammatory process. Finally, possible cytotoxicity on human gastric adenocarcinoma cells (AGS) was tested. After 24 h, the cytotoxic effect of both extracts was concentration-dependent in the same concentration range (the *A. equina* extract was more cytotoxic, IC_50_ = 0.365 mg/mL): homarine was cytotoxic as well, causing decreased cell viability by 27.57% ± 5.42% at the highest concentration tested of 1 mg/mL). At the 48 and 72 h treatments, results were similar to the 24 h effects. Both extracts activated caspase 3, while homarine did not. On the contrary, caspase-4 activity was increased after incubation with the aqueous extracts of *A. sulcata* and homarine, while not by the *A. equine* extract. Altogether, these data suggest a non-classical mechanism of apoptosis mediated by caspase-4 and -3 in human gastric cells. The study gave new insights on the toxicity and biological potential of the two sea anemones, which are increasingly used in human nutrition.

Anti-inflammatory activity was also observed for other sea anemones. Sea anemones are rich sources of Kunitz-type polypeptides, which can have proteinase inhibitory, Kv channels toxicity, analgesic, antihistamine and anti-inflammatory activities. In 2015, Gladkikh and co-workers [125] isolated two Kunitz-type inhibitors from the sea anemone *H. crispa*, named HCRG1 and HCRG2. Both showed anti-inflammatory activity, reducing the secretion of the pro-inflammatory mediators tumor necrosis factor-α (TNF-α), interleukin 6 (IL-6) and proIL-1β expression in lipopolysaccharide (LPS)-activated macrophages. TNF-α and IL-6 reduction was evaluated by enzyme-linked immunosorbent assay (ELISA) and HCRG1 and HCRG2 were active when tested at both 1, 3 and 10 μM. The proIL-1β expression reduction was evaluated by Western blotting and HCRG1 and HCRG2 were active when tested at both 1 and 10 μM. Similarly, the HCGS 1.20 recombinant polypeptide from the same sea anemone species showed anti-inflammatory activity by inhibiting the histamine-induced increase in the concentration of calcium ions in mouse bone marrow-derived macrophages and the lipopolysaccharide-stimulated increase in the concentration of nitric oxide in RAW 264.7 mouse macrophages [131]. Successively, two recombinant peptides, the Kunitz-type serine protease inhibitors rHCGS1.19 and rHCGS1.36, from *H. crispa* showed antihistamine activity at 10 μM, inhibiting an increase in the calcium ion concentration in murine bone marrow-derived macrophages elicited by histamine at 62.2 and 84.0%, respectively [125,132]. HCGS1.36 and HCGS1.10 have also shown analgesic effects in the thermal pain stimulation model (at a concentration of 0.5 mg/kg) [130].

Sea anemones have been shown to be also excellent sources of human pancreatic α-amylase inhibitors with possible applications in the control of blood sugar levels in the management of diabetes mellitus patients. In particular, helianthamide was isolated from the Caribbean Sea anemone *Stichodactyla helianthus* and showed to adopt a β-defensin fold and bind into and across the amylase active site (with Ki = 10 pM) [152]. Magnificamide, recently isolated from sea anemone *Heteractis magnifica*, shared 84% sequence identity with helianthamide and inhibited porcine pancreatic and human saliva α-amylases with Ki’s equal to 0.17 ± 0.06 nM and 7.7 ± 1.5 nM, respectively, showing to be another potential drug candidate for diabetes treatment [139]

Venom proteins identified in the cubomedusa *Chironex fleckeri*, CfTX-1, CfTX-2, CfTX-A and CfTX-B, showed possible cardiovascular and cytolytic applications [18]. In particular, CfTX-1/2 (25 µg kg^−1^) had effects on the cardiovascular system of anesthetized rats, while CfTX-A/B were less active. In addition, CfTX-A/B had a hemolytic activity 30 times strong than CfTX-1/2.

Recently, a new metalloproteinase was identified and partially purified from *Rhizostoma pulmo* (Scyphozoa) [51]. This metalloproteinase showed significant hemolytic activity against human red blood cells and a strong proteolytic activity for substrates like (azo) casein and gelatin [51].

With the urgent need to discover and develop new antibiotics, over the past few decades, several studies have explored the antimicrobial/antibiotic properties of cnidarian extracts [208]. Mariottini and Grice [208] recently reviewed the antimicrobial compounds from both marine and freshwater Cnidaria. Their study highlighted the presence of several active compounds. However, due to sampling difficulties and extracting low amounts, very few proceeded in pre-clinical evaluation and no one reached the market.

In addition to these therapeutic possible applications, it is important to remind the reader that various Cnidaria species, mainly scyphomedusae, are a common ingredient in the Eastern cooking tradition and suggested in Western countries as an alternative/integrative source of food thanks to their rich composition of proteins and fatty acids (see review of Merquiol et al. [17]). Scyphomedusae have been also suggested as providing a contribution to the cosmeceutical and biomaterial industries (for more details, see the reviews by [17,209]). However, at present these remain potential biotechnological applications that still need to be developed.

## 5. Conclusions

Despite the fact that cnidarian venoms are known for their often-lethal effects, already since ancient times, only a few groups of cnidarians have been studied in detail to define their composition. Our review highlights that most attention related to chemical defences in Cnidaria has been given to identify the compounds in the venom extracted from Anthozoa, Cubozoa and Hydrozoa. This attention is likely due to the fact that most Cubozoa and some species of Hydrozoa (for example *Physalia physalis*) not only induce painful stings, but their venom can have lethal effects on humans. Nevertheless, Cnidaria, such as sea anemones, contain compounds within their venoms that are not readily lethal for humans, but are indeed helpful to highlight the processes in healthy and unhealthy cells. The understanding of these mechanisms promotes a better knowledge of the physiological processes and stimulates the application of compounds extracted from cnidarian venom in biotechnology, particularly in the biomedical field.

The use of omics approaches has been beneficial to the progress of research in this field [210]. Omics approaches have helped to detect and identify compounds that may have been overlooked using non-omics techniques. However, omics have been used in a very limited number of studies at present. Nevertheless, Cnidaria may represent an interesting source of bioactive peptides for new drug development. However, Cnidaria toxin exploitation in the pharmaceutical industry has been slowed down by sourcing problems, as the sampling is not considered eco-friendly and has limited high-throughput discovery and isolation. Recent advancements in multiple “omic” technologies, including genomics, transcriptomics, proteomics and metabolomics, coupled with advanced bioinformatics, have opened the way for large-scale discovery of new molecules from marine organisms. High-throughput methods combining chemical analyses with omic profiling can provide a holistic overview of Cnidaria complexity, giving new opportunities to discover new peptides and other bioactive metabolites with potential applications for the treatment of human diseases.

## Figures and Tables

**Table 1:** The main five classes of the phylum Cnidaria with their life stages and cnida traits.

Class	Life Stage	Cnida Type	Reference
Hydrozoa	Alternance polyp/medusa	Nematocyst	[3,4]
Scyphozoa	Alternance polyp/medusa	Nematocyst	[3,4]
Cubozoa	Alternance polyp/medusa	Nematocyst	[3,4]
Anthozoa	Polyp only	Nematocysts	[3,4]
		Spirocyst (Zoantharia only)	[4]
		Ptychocytes (Ceriantharia only)	[5]
Staurozoa	Alternance polyp/medusa	Nematocysts	[6]

**Table 2:** Toxins identified in cnidarian species, including their molecular mass (kDa) and biological activity. N/A means not available. TRPV1 stands for Vanilloid Receptor 1, TRPA1 stands for transient receptor potential ankyrin 1 ion channel and ASIC stands for acid-sensing ion channel.

Species	Compound	Molecular Mass (kDa)	Biological Activity	References
Hydrozoa				
*Hydra magnipapillata*	CqTX-A	~40	Cardiovascular, hemolytic	[18]
	HALT-1	N/A	Hemolytic, cytolysis	[19]
	HALT1–HALT7	N/A	Cytolytic	[20]
*H. viridissima*	Hydralysin	27	Neurotoxic, cytolytic, paralytic	[21]
*Millepora platyphylla*	Milleporin-1	30–34	Cytolytic, hemolytic	[22]
*Olindias sambaquiensis*	Oshem1	3.013	Cytolytic, hemolytic, myonecrotic, and cytotoxic	[23]
	Oshem2	3.376	Cytolytic	[23]
	Metalloproteinases	N/A	Cytolytic, neurotoxic	[24]
*Physalia physalis*	Phospholipase A_2_	N/A	N/A	[25]
	Phospholipase B	N/A	N/A	[25]
	Collagenase	25	Cytotoxic, hemolytic	[26]
	Elastases	N/A	Musculotoxic, cytolytic, hemolytic	[27]
	PpV19.3	4.72	Neurotoxic, cardiotoxic	[28]
	PpV9.4	0.55	Hemolytic	[28]
	P3	85	Neurotoxic	[29]
	P1	220	Neurotoxic	[30]
	Physalitoxin	220	Hemolytic	[31]
	DNase	75	Cytolytic	[32]
	Histamine	N/A	N/A	[33]
*Tubularia larynx*	Phospholipase A_2_	N/A	Cytolytic, hemolytic	[15]
Scyphozoa				
*Aurelia aurita*	Phospholipase A_2_	N/A	Cytolytic	[15,34]
	Proteolytic enzymes	N/A	Hemolytic, neurotoxic, myotoxic, local skin irritation	[35]
	Tetramine and unidentified protein	N/A	Dermotoxic, temporary paralysis, edema	[36]
	Metalloproteinases	N/A	Gelatinolytic, caseinolytic, fibrinolytic	[37]
	Aurelin	4.30	Antimicrobial, neurotoxic (voltage-gated potassium channel inhibitor)	[38]
*Cassiopea andromeda*	Phospholipase A_2_	N/A	Hemolytic, dermonecrotic, local skin irritation	[34]
*C. xamancha*	Phospholipase A_2_	N/A	Hemolytic, dermonecrotic, local skin irritation	[34]
*Chrysaora hysoscella*	Cationic protein	N/A	Dermotoxic, cytotoxic	[39]
*C. quinquecirrha*	DNase	110	Dermonecrotic, cytotoxic	[26]
	Acid protease	120–150	N/A	[26]
	Metallopeptidase	100	N/A	[26]
	Collagenase	N/A	N/A	[26]
*Cyanea capillata*	Basic protein(s)	70	Cardiotoxic, dermonecrotic, musculotoxic	[26,40]
	CcTX-1	31.173	Cytotoxic	[41]
	CcNT	8.22	Neurotoxic	[42]
	Phospholipase A_2_	N/A	Cytolytic, cytotoxic, hemolytic	[15,43]
*C. lamarckii*	ClGP-1	27	Cytotoxic	[43]
	Phospholipase A_2_	N/A	Cytolytic, cytotoxic, hemolytic	[43]
*C. nozakii*	Metalloproteinases	N/A	Gelatinolytic, caseinolytic, fibrinolytic	[37]
*Nemopilema nomurai*	Metalloproteinases	28–36	Gelatinolytic, caseinolytic, fibrinolytic	[37]
		20–40/10–15	Cytotoxic, hemolytic	[44]
*Pelagia noctiluca*	Proteinaceous macromolecules	44–66	Hemolytic, cytotoxic, dermonecrotic, hemolytic, local tissue damage	[45,46,47,48,49]
*Phyllorhiza punctata*	Phospholipase A_2_	N/A	Neurotoxic	[50]
*Rhizostoma pulmo*	Rhizoprotease	95	Proteolytic, hemolytic	[51]
	Rhizolysin	260	Hemolytic	[52]
		N/A	Cytotoxic, hemolytic	[53]
*Rhopilema esculentum*	Metalloproteinases	N/A	Gelatinolytic, caseinolytic, fibrinolytic	[37]
	Hyaluronidase	55–95	Degradation of extracellular matrix components	[37]
		N/A	Proteolytic, cytotoxic, hemolytic	[54,55]
*R. nomadica*	Phospholipase A_2_	N/A	Hemolytic	[56]
	Serine protease	N/A	Local skin damage	[57]
*Rhopilema* spp.	Phospholipase A_2_	N/A	Hemolytic	[58]
*Stomolophus meleagris*	SmP90	90	Radical scavenging	[59]
	Phospholipase A_2_	N/A	Cytotoxic, cytolytic, hemolytic, local tissue damage	[59]
	218 toxins including C-lectin and metalloprotease	N/A	Voltage-gated potassium channel inhibitor	[59]
Cubozoa				
*Alatina moseri*	CaTX-A	43	Hemolytic	[18]
*Akatina alata*	CAH1	42	Hemolytic	[60]
	CaTX-A	43	Hemolytic	[61]
	CaTX-B	45	Hemolytic	[61]
*C. marsupialis*	Haemolysin	102–107	Hemolytic	[62]
	CmHl1	139	Cytolytic	[63]
	CmHl5	220	Cytolytic	[63]
	CmH17	139	Cytolytic	[63]
	CmNt	120	Neurotoxic	[63]
*C. rastonii*	Phospholipase A_2_	N/A	Cytolytic	[15]
	CrTX-I	N/A	Hemolytic	[64]
	CrTX-II	N/A	Hemolytic	[64]
	CrTX-III	N/A	Hemolytic	[64]
	CrTX-A	43	Cutaneous inflammation of human skin	[61]
	CrTX-B	46	N/A	[61]
*Carukia barnesi*	Phospholipase A_2_	N/A	Cytolytic, hemolytic	[15]
	CbTX-I	21.67	Neurotoxic	[65]
	CbTX-II	18.16	Neurotoxic	[65]
*Chironex fleckeri*	Phospholipase A_2_	N/A	Cytolytic, hemolytic	[15]
	Metalloproteinases	17–130	N/A	[66]
	CfTX-1	43	Cardiotoxic, cytotoxic, dermonecrotic, lethal	[67]
	CfTX-2	45	Cardiotoxic, cytotoxic, dermonecrotic, lethal	[67]
	CfTX-A	40	Hemolytic	[18]
	CfTX-B	42	Hemolytic	[18]
	CfTX-Bt	31.29	N/A	[18]
*Chiropsalmus quadrigatus*	CqTX-A	44	Hemolytic, neurotoxic, myotoxic	[58,68,69]
*Malo kingi*	MkTX-A; MkTX-B	43–45	Dermonecrotic, inflammatory	[65]
		43–45	Dermonecrotic, inflammatory	[49]
Anthozoa (Hexacorallia)				
*Acropora* spp.	Phospholipase A_2_	N/A	Catalytic	[15]
*Actinia australis*	Phospholipase A_2_	N/A	Catalytic	[15]
*Actinia equina*	AeI	N/A	Lethal activity to crabs, Type I sodium channel toxin	[70]
	AEPI-I, II, III and IV	6.2–7	Kunitz-type toxins	[71]
	Equinatoxin-I, II and III	19	Cytolytic and hemolytic	[72]
	Equinatoxin-IV	N/A	Hemolytic	[73]
	Equinatoxin-V	N/A	N/A	[74]
	Acrorhagin I and II	N/A	Lethal activity to crabs	[75]
	Acrorhagin Ia and IIa	N/A	N/A	[75]
*Actinia fragacea*	Fragaceatoxin C	20	Lytic	[76]
*Actinia tenebrosa*	Tenebrosin-A, B and C	19–20	Hemolytic	[77]
*Actinia villosa*	Avt-I	19	Hemolytic	[78]
	Avt-II	N/A	Cytolysis	[78,79]
	AvTX-60A	60	Fatal toxicity to mice	[80]
	Avt120	120	Lethal activity to mice, cytotoxic	[81]
*Adamsia palliata*	AcPLA2	13.5	Phospholipase A2 catalytic activity	[82]
*Adamsia carciniopados*	Phospholipase A2	N/A	Phospholipase A2 catalytic activity	[15]
*Anemonia erythraea*	AETX-I	5	Crab lethality, Type I sodium channel toxin	[83]
	AETX-K	~4	Type I potassium channel toxin	[84]
	AETX-II	6.5	Crab lethality	[83]
	AETX-III	6.6	Crab lethality	[83]
*Anemonia sulcata*	Toxin I	N/A	Type I sodium channel toxin	[85]
	ATX-II	5	Cardiotoxic action, Type I sodium channel toxin	[62,85]
	Toxin III	2.7	N/A	[86]
	ATX-V	N/A	Type I sodium channel toxin	[87]
	SA5 II	N/A	Kunitz-type proteinase toxin	[88]
	Kalicludin 1, 2, 3	~6.7	Type II potassium channel toxin, Kunitz inhibitors	[89]
	BDS-I and BDS-II	4.7	Inactivating Kv3.4 channel	[78]
	Kaliseptine	3.8	Potassium channel toxin	[89]
*Anthopleura asiatica*	Bandaporin	20	Hemolytic	[90]
*Anthopleura elegantissima*	Anthopleurin-C	N/A	Cardiotoxic to rats, Type I sodium channel toxin	[91]
	APE 1-1, 1-2, 2-1, 2-2, 3, 4 and 5-3	5	Crab paralysis, Type I sodium channel toxin	[92]
	APET x2	4.6	ASIC3 inhibition (acting on channel external side)	[93]
*Anthopleura fuscoviridis*	AFT-I and II	N/A	Type I sodium channel toxins	[94]
*Anthopleura xanthogrammica*	Anthopleurin-A and B	N/A	Cardiotoxic to rats, Type I sodium channel toxin	[91]
	Toxin PCR1-2, 2-1, 2-5, 2-10, 3-6, 3-7, 3-3,4	N/A	Type I sodium channel toxin	[95]
	AXPI-I and II	N/A	Kunitz-type inhibitor	[96]
*Anthopleura* spp.	Hk2a, Hk7a, Hk8a, Hk16a	~5	Rat heart stimulation, Type I sodium channel toxins	[97]
*Bolecera tuedia*	Phospolipase A2	N/A	Phospholipase A2 catalytic activity	[15]
*Bunodosoma caissarum*	Bc-I, II and III	N/A	Toxic on crustacean nerves, Type I sodium channel toxin	[98]
	Bc-IV	~5	Weak-paralyzing action in swimming crabs	[99]
	BcPLA(2)1	~5	Renal function and induced insulin secretion in conditions of high glucose concentration	[100]
	BcsTx3	5.71	Potassium channel inhibition	[101]
*Bunodosoma granulifera*	Bg II and III	N/A	Neurotoxic on mice, Type I sodium channel toxins	[102]
	Bg toxin	~4	Type I potassium channel toxin	[103]
	Granulitoxin (GRX)	~5	Severe neurotoxic effects such as circular movements, aggressive behavior, dyspnea, tonic-clonic convulsion and death in mice	[104]
*Bunodosoma cangicum*	Cangitoxin	5	Type I sodium channel toxin	[105]
	Cangitoxin II and III (CGTX-II and CGTX-III)	~5	Type I sodium channel toxin	[106]
	Bcg 25.96, 28.19, 30.24	~4	Type I potassium channel toxin, Neurotoxic to crabs	[105]
	Bcg 31.16, 28.78, 25.52, 29.21	4–5	May target different types of ion channels, Neurotoxic to crabs	[105]
	Bcg 21.75, 23.41, 21.00	3	Possible new class of potassium channel blockers, Neurotoxic to crabs	[105]
*Calliactis parasitica*	Calitoxin 1	~5	Increased transmitter release, causing repetitive firing of the axons in neuromuscular preparation of crustaceans	[107]
	Calitoxin 2	N/A	N/A	[108]
*Condylactis gigantea*	CgNa	~5	Type I sodium channel toxin	[109]
*Condylactis passiflora*	CpI, II and III	N/A	Lethal activity against crabs, Type I sodium channel toxin	[110]
*Cryptodendrum adhaesivum*	Ca I	N/A	Type II sodium channel toxin	[111]
*Dendronephthya* spp.	Phospholipase A2	N/A	Phospholipase A2 catalytic activity	[15]
*Halcurias carlgreni*	Halcurin	~5	N/A	[112]
*Heteractis crispa (=Radianthus crispus = R. macrodactyla)*	Neurotoxin I	N/A	Type II sodium channel toxin	[113]
	Neurotoxin II	N/A	Type II sodium channel toxin	[114]
	Neurotoxin III	N/A	Type II sodium channel toxin	[115]
	Neurotoxin IV and V	N/A	Type II sodium channel toxins	[116]
	Rc I	N/A	Lethal against crabs, Type I sodium channel toxin	[117]
	Kunitz-type Trypsin inhibitor IV	N/A	Kunitz-type inhibitor	[118]
	Actinoporin RTX-A	N/A	Hemolytic	[119]
	Actinoporin RTX-S II	19	Hemolytic	[120]
	HCIQ2c1	6	Neuroprotective, Kunitz-type inhibitor	[121]
	Hcr 1b-1	4.5	ASIC3 channel inhibitor	[122]
	Hcr1b-2, Hcr1b-3, Hcr1b-4	5	Neurotoxic (ASIC1 channel inhibitors)	[123]
	HCRG1, HCRG2	6	Anti-inflammatory, Kunitz-type inhibitors	[124]
	InhVJ	6	Kunitz-type inhibitor	[125]
	HGRC21	6	Analgesic, inhibitor of TRPV1, Kunitz-type inhibitor	[126]
	APHC1, APHC2, APHC3	6	Analgesic (TRPV1 modulation)	[127,128,129]
	HCGS 1.10	6	Analgesic	[130]
	HCGS 1.20	6	Anti-inflammatory	[131]
	rHCGS1.19 and rHCGS1.36	6	Antihistamine, Kunitz-type serine protease inhibitors	[132]
*Heteractis magnifica (=Radianthus magnifica = R. paumotensis = R. ritteri)*	Magnificalysins I and II	~19	Hemolytic and lethal activity on mice	[133]
	HMGS1	6	Kunitz-type inhibitor	[134]
	HMgIII		Hemolytic and cytolytic	[135]
	HmK	~4	Potassium channel inhibitor	[136]
	RpI, RpII, RpIII, and RpIV	~5	Toxic in mice and crabs, Type II sodium channel toxins	[137]
	HMIQ3c1	N/A	Neuroprotective (Kunitz-type inhibitor)	[138]
	Magnificamide	4.7	Alpha-amylase inhibitor	[139]
*Heterodactyla hemprichi*	δ-TLTX-Hh1a and δ-TLTX-Hh1c	N/A	Lethal on crabs, Type II sodium channel toxin	[111]
*Metridium senile*	Metridin	3.97	Hemolytic	[140]
	Phospholipase A2	N/A	Phospholipase A2 catalytic activity	[15]
	Ms 9a-1	3.6	Analgesic by potentiating TRPA1	[141]
*Nematostella vectensis*	Nv1116.25.1, 116.27.1, 116.28.1, 116.37.1, 116.39.1, 116.40.1, 116.41.1, 116.45.1	N/A	Type II sodium channel toxins	[142]
	Nv4 – Nv8	N/A	Sodium channel inhibitors	[143]
	NEP1 – NEP20	N/A	N/A	[144]
*Oulactis orientalis*	Actinoporin Or-A and Or-G	~18	Cytolytic	[145]
	NEP1 to NEP 20	N/A	N/A	[144]
*Parasicyonis actinostoloides*	PA-TX	N/A	N/A	[146]
*Phyllodiscus semoni*	PsTX-115	N/A	Renal injury	[147]
	PsTX-60A; PsTX-60B	60	Cytolytic, hemolytic	[148]
*Phymanthus crucifer*	PhcrTx1	3.47	ASIC inhibitor	[149]
*Pocillopora damicornis*	Phospholipase A2	N/A	Phospholipase A2 catalytic activity	[15]
*Sagartia rosea*	Cytolysin Src-I	19.6	Cytolysis	[150]
*Sarcophyton elegans*	Phospolipase A2	N/A	Phospholipase A2 catalytic activity	[15]
*Stichodactyla helianthus*	Sh1	~5	Neurotoxic on crabs, Type II sodium channel inhibitor	[151]
	Helianthamide	4.7	Alpha-amylase inhibitor	[152]
	SHPI-1	~6	Kunitz-type proteinase inhibitor	[153]
	ShK	~5	Potassium channel inhibitor	[154]
	Sticholysin-I and II	~19	Hemolytic	[155]
*Stoichactis* sp.	Phospholipase A2	N/A	Phospholipase A2 catalytic activity	[15]
*Stichodactyla gigantea*	Gigantoxins I-III	N/A	Crab toxicity, Sodium channel inhibitors	[156]
*Stichodactyla haddoni*	SHTX I–III	~7	Crab-paralyzing activity, SHTX-III is a Kunitz-type proteinase inhibitor	[157]
	SHTX IV	~5	Crab lethality, Type II sodium channel toxin	[157]
	EGF-like peptide SHTX-5	N/A	N/A	[157]
*Thalassianthus aster*	Ta I3,8-7,6	N/A	Type II sodium channel toxin	[111]
*Urticina coriacea*	U1	N/A	Hemolytic, cytotoxic	[124]
	U2	3–10.5	Analgesic (ASIC1 channel inhibitor)	[124]
*Urticina crassicornis*	Uc-I	30	Cytolysis	[158]
*Urticina eques*	Ueq 12-1	4.79	Antibacterial, analgesic by potentiating TRPA1	[159]
*Urticina grebenyi*	UGR9a-1	N/A	Analgesic, ASICS channel inhibitor	[160]
*Urticina piscivora*	Up-I	28	Hemolytic	[161]
*Virgularia nidularis*	Phospholipase A2	N/A	Phospholipase A2 catalytic activity	[15]
*Anthozoa (Octocorallia)*				
*Alcyonum digitatum*	Phospholipase A2	N/A	Phospholipase A2 catalytic activity	[15]
*Paramuricea* spp.	Phospholipase A2	N/A	Phospholipase A2 catalytic activity	[15]
*Sinularia flexibilis*	Phospholipase A2	N/A	Phospholipase A2 catalytic activity	[15]

**Table 3:** Cnidaria genome deposited in Genbank, their Assembly Accession Numbers (accessed on 8 July 2020) and references. N.A. stands for not available.

Species	Accession Number	Reference
Hydrozoa		
*Clytia hemisphaerica*	GCA_902728285.1	N.A.
*Craspedacusta sowerbii*	GCA_003687565.1	N.A.
*Hydra oligactis*	GCA_004118135.1	[176]
*Hydra viridissima*	GCA_004118115.1	[176]
*Hydra vulgaris*	GCA_000219015.1	[177]
*Hydra vulgaris*	GCA_000004095.1	[177]
Scyphozoa		
*Aurelia aurita*	GCA_004194415.1	N.A.
*Aurelia aurita complex* sp. *pacific*	GCA_004194395.1	N.A.
*Aurelia coerulea*	GCA_011634815.1	N.A.
*Cassiopea xamachana*	GCA_900291935.1	N.A.
*Chrysaora quinquecirrha*	GCA_012295145.1	N.A.
*Chrysaora chesapeakei*	GCA_011763395.1	N.A.
*Chrysaora fuscescens*	GCA_009936425.1	N.A.
*Nemopilema nomurai*	GCA_003864495.1	N.A.
*Rhopilema esculentum*	GCA_013076305.1	N.A.
*Sanderia malayensis*	GCA_013076295.1	N.A.
Cubozoa		
*Alatina alata*	GCA_008930755.1	[178]
*Alatinida* sp. Z8VKAUB7J3	GCA_010016025.1	N.A.
*Carybdea marsupialis* auct. non (Linnaeus, 1758)	GCA_010016065.1	N.A.
*Morbakka virulenta*	GCA_003991215.1	N.A.
Anthozoa		
*Actinia equina*	GCA_011057435.1	[179]
*Actinia tenebrosa*	GCA_009602425.1	[180]
*Acropora digitifera*	GCA_000222465.2	[181]
*Acropora millepora*	GCF_004143615.1	[182]
*Anemonia viridis*	GCA_900234385.1	N.A.
*Dendronephthya gigantea*	GCA_004324835.1	[183]
*Exaiptasia diaphana*	GCA_001417965.1	[184]
*Heteractis magnifica*	GCA_011763375.1	N.A.
*Montipora capitata*	GCA_006542545.1	[185]
*Nematostella vectensis*	GCA_000209225.1	[186]
*Orbicella faveolata*	GCA_002042975.1	N.A.
*Orbicella faveolata*	GCA_001896105.1	[187]
*Phymanthus crucifer*	GCA_009858155.1	N.A.
*Pocillopora damicornis*	GCA_003704095.1	[188]
*Porites rus*	GCA_900290455.1	N.A.
*Renilla reniformis*	GCA_900177555.1	N.A.
*Stichodactyla mertensii*	GCA_011800005.1	N.A.
*Stylophora pistillata*	GCA_002571385.1	[189]
Staurozoa		
*Calvadosia cruxmelitensis*	GCA_900245855.1	N.A.
Myxozoa		
*Enteromyxum leei*	GCA_001455295.2	[190]
*Henneguya salminicola*	GCA_009887335.1	N.A.
*Kudoa iwatai*	GCA_001407235.2	[190,191]
*Kudoa iwatai*	GCA_001407335.1	[191]
*Myxobolus squamalis*	GCA_010108815.1	N.A.
*Sphaeromyxa zaharoni*	GCA_001455285.1	[191]
*Thelohanellus kitauei*	GCA_000827895.1	[192]

## References

[1] Arai M.N. (1997). A Functional Biology of Scyphozoa.

[2] Technau U., Steele R.E. (2012). Evolutionary crossroads in developmental biology. Cnidaria. Dev..

[3] Mariscal R.N., Lenhoff K., Muscatine L., Marian E., Davis L. (1974). Nematocysts. Coelenterate Biology.

[4] Östman C. (2000). A guideline to nematocyst nomenclature and classification, and some notes on the systematic value of nematocysts. Sci. Mar..

[5] Mariscal R.N., Conklin E.J., Bigger C.H. (1977). The ptychocyst, a major new category of cnida used in tube construction by a cerianthid anemone. Biol. Bull..

[6] Larson R.J., Fautin D.G. (1989). Stauromedusae of the genus *Manania* (=*Thaumatoscyphus*) (Cnidaria, Scyphozoa) in the Northeast Pacific, including descriptions of new species *Manania gwilliami* and *Manania handi*. Can. J. Zool..

[7] Ames C.L., Klompen A.M.L., Badhiwala K., Muffett K., Reft A.J., Kumar M., Janssen J.D., Schultzhaus J.N., Field L.D., Muroski M.E. (2020). Cassiosomes are stinging-cell structures in the mucus of the upside-down jellyfish *Cassiopea xamachana*. Commun. Biol..

[8] Fautin D. (2009). Structural diversity, systematics, and evolution of cnidae. Toxicon.

[9] Moran Y., Genikhovich G., Gordon D., Wienkoop S., Zenkert C., Ôzbek S., Technau U., Gurevitz M. (2012). Neurotoxin localization to ectodermal gland cells uncovers an alternative mechanism of venom delivery in sea anemones. Proc. Rotal Soc. Lond. (Part B).

[10] Jouiaei M., Yanagihara A., Madio B., Nevalainen T.J., Alewood P.F., Fry B. (2015). Ancient venom systems: A review on Cnidaria toxins. Toxins.

[11] Frazão B., Vasconcelos V., Antunes A. (2012). Sea anemone (Cnidaria, Anthozoa, Actiniaria) toxins: An overview. Mar. Drugs.

[12] Mariottini G.L. (2014). Hemolytic venoms from marine cnidarian jellyfish—An overview. J. Venom Res..

[13] Mariottini G.L., Pane L. (2010). Mediterranean jellyfish venoms: A review on scyphomedusae. Mar. Drugs.

[14] Mariottini G.L., Pane L. (2014). Cytotoxic and cytolytic cnidarian venoms. A review on health implications and possible therapeutic applications. Toxins.

[15] Nevalainen T.J., Peuravuori H.J., Quinn R.J., Llewellyn L.E., Benzie J.A.H., Fenner P.J., Winkel K.D. (2004). Phospholipase A2 in Cnidaria. Comp. Biochem. Physiol. Part B Biochem. Mol. Biol..

[16] Moran Y., Gordon D., Gurevitz M. (2009). Sea anemone toxins affecting voltage-gated sodium channels—Molecular and evolutionary features. Toxicon.

[17] Merquiol L., Romano G., Ianora A., D’Ambra I. (2019). Biotechnological applications of scyphomedusae. Mar. Drugs.

[18] Brinkman D.L., Konstantakopoulos N., McInerney B.V., Mulvenna J., Seymour J.E., Isbister G.K., Hodgson W.C. (2014). *Chironex fleckeri* (box jellyfish) venom proteins: Expansion of a cnidarian toxin family that elicits variable cytolytic and cardiovascular effects. J. Biol. Chem..

[19] Glasser E., Rachamim T., Aharonovich D., Sher D. (2014). *Hydra* actinoporin-like toxin-1, an unusual hemolysin from the nematocyst venom of *Hydra magnipapillata* which belongs to an extended gene family. Toxicon.

[20] Yap W.Y., Tan K.J.S.X., Hwang J.S. (2019). Expansion of *Hydra* actinoporin-like toxin (HALT) gene family: Expression divergence and functional convergence evolved through gene duplication. Toxicon.

[21] Zhang M., Fishman Y., Sher D., Zlotkin E. (2003). Hydralysin, a novel animal group-selective paralytic and cytolytic protein from a noncnidocystic origin in *Hydra*. Biochemistry.

[22] Radwan F.F.Y., Aboul-Dahab H.M. (2004). Milleporin-1, a new phospholipase A2 active protein from the fire coral *Millepora platyphylla* nematocysts. Comp. Biochem. Physiol. C Toxicol. Pharmacol..

[23] Haddad V., Zara F., Marangoni S., Toyama D., Souza A., Oliveira S., Toyama M. (2014). Identification of two novel cytolysins from the hydrozoan *Olindias sambaquiensis* (Cnidaria). J. Venom. Anim. Toxins Incl. Trop. Dis..

[24] Weston A.J., Chung R., Dunlap W.C., Morandini A.C., Marques A.C., Moura-da-Silva A.M., Ward M., Padilla G., da Silva L.F., Andreakis N. (2013). Proteomic characterisation of toxins isolated from nematocysts of the South Atlantic jellyfish *Olindias sambaquiensis*. Toxicon.

[25] Stillway L.W., Lane C.E. (1971). Phospholipase in the nematocyst toxin of *Physalia physalis*. Toxicon.

[26] Burnett J.W., Calton G.G. (1987). Venomous pelagic coelenterates chemistry toxicology immunology and treatment of their stings. Toxicon.

[27] Werb Z., Banda M.J., McKerrow J.H., Sandhaus R.A. (1982). Elastases and elastin degradation. J. Investig. Dermatol..

[28] Dìaz Garcìa C., Fuentes-Silva D., Sanchez-Soto C., Domìnguez Pàrez D., Garcìa-Delgado N., Varela C., Mendoza-Hernàndez G., Rodrìguez-Romero A., Castañeda O., Hiriart M. (2012). Toxins from *Physalia physalis* (Cnidaria) raise the intracellular Ca2+ of beta-cells and promotei Insulin secretion. Curr. Med. Chem..

[29] Mas R., Menéndez R., Garateix A., Garcia M., Cha M. (1989). Effects of a high molecular weight toxin from *Physalia physalis* on glutamate responses. Neuroscience.

[30] Menéndez R., Mas R., Garateix A., Garcia M., Chavez M. (1990). Effects of a high molecular weight polypeptidic toxin from *Physalia physalis* (Portuguese man-of-war) on cholinergic responses. Comp. Biochem. Physiol. Part C Comp. Pharmacol..

[31] Tamkun M.M., Hessinger D.A. (1981). Isolation and partial characterization of a hemolytic and toxic protein from the nematocyst venom of the Portuguese man-of-war, *Physalia physalis*. Biochim. Biophys. Acta (BBA) Protein Struct..

[32] Neeman I., Calton G.J., Burnett J.W. (1980). Purification and characterization of the endonuclease present in *Physalia physalis* venom. Comp. Biochem. Physiol. Part B Comp. Biochem..

[33] Cormier S.M. (1981). *Physalia* venom mediates histamine release from mast cells. J. Exp. Zool..

[34] Radwan F.F.Y., Burnett J.W., Bloom D.A., Coliano T., Eldefrawi M.E., Erderly H., Aurelian L., Torres M., Heimer-de la Cotera E.P. (2001). A comparison of the toxinological characteristics of two *Cassiopea* and *Aurelia* species. Toxicon.

[35] Bayazit V. (2004). Cytotoxic effects of some animal and vegetable extracts and some chemicals on adenohypophyse carcinoma, kidney adenocarcinoma and skin carcinoma cells. J. Med. Sci..

[36] Burnett J.W., Calton G.J., Larsen J.B. (1988). Significant envenomation by *Aurelia aurita*, the moon jellyfish. Toxicon.

[37] Lee H., Jung E.-S., Kang C., Yoon W.D., Kim J.-S., Kim E. (2011). Scyphozoan jellyfish venom metalloproteinases and their role in the cytotoxicity. Toxicon.

[38] Ovchinnikova T.V., Balandin S.V., Aleshina G.M., Tagaev A.A., Leonova Y.F., Krasnodembsky E.D., Men’shenin A.V., Kokryakov V.N. (2006). Aurelin, a novel antimicrobial peptide from jellyfish *Aurelia aurita* with structural features of defensins and channel-blocking toxins. Biochem. Biophys. Res. Commun..

[39] Kokelj F., Del Negro P., Tubaro A. (1989). Dermossicità da Chrysaora hysoscella. G. Ital. Dermatol. Venereol..

[40] Walker M.J.A. (1977). Pharmacological and biochemical properties of a toxin containing material from the jellyfish, *Cyanea capillata*. Toxicon.

[41] Lassen S., Helmholz H., Ruhnau C., Prange A. (2011). A novel proteinaceous cytotoxin from the northern Scyphozoa *Cyanea capillata* (L.) with structural homology to cubozoan haemolysins. Toxicon.

[42] Lassen S., Wiebring A., Helmholz H., Ruhnau C., Prange A. (2012). Isolation of a Na_v_ channel blocking polypeptide from *Cyanea capillata* medusae—A neurotoxin contained in fishing tentacle isorhizas. Toxicon.

[43] Helmholz H., Ruhnau C., Schütt C., Prange A. (2007). Comparative study on the cell toxicity and enzymatic activity of two northern scyphozoan species *Cyanea capillata* (L.) and *Cyanea lamarckii* (Péron & Léslieur). Toxicon.

[44] Kang C., Munawir A., Cha M., Sohn E.-T., Lee H., Kim J.-S., Yoon W.D., Lim D., Kim E. (2009). Cytotoxicity and hemolytic activity of jellyfish *Nemopilema nomurai* (Scyphozoa: Rhizostomeae) venom. Comp. Biochem. Physiol. Part C Toxicol. Pharmacol..

[45] Quadrifoglio F., Avian M., Del Negro P., Princi T., Scuka M., Gavinelli E., Rottini Sandrini L. (1986). Nematocisti e tossine di *Pelagia noctiluca* (Forsskål). Nova Thalass..

[46] Salleo A., Calabrese L., Barra D., La Spada G. (1986). Characterization of protein components of the capsule fluid ad of the capsule wall of the nematocysts of *Pelagia noctiluca*. Nova Thalass..

[47] Scarpa C., Kokelj F., Del Negro P., Tubaro A. (1987). Valutazione dell’effetto irritante sulla cute umana di una preparazione di nematocisti di *Pelagia noctiluca*. Ann. It. Derm. Clin. Sper..

[48] Mariottini G.L., Giacco E., Pane L. (2008). The mauve stinger *Pelagia noctiluca* (Forsskal, 1775). Distribution, ecology, toxicity and epidemiology of stings. A review. Mar. Drugs.

[49] Morabito R., Marino A., La Spada G., Pane L., Mariottini G.L. (2015). The venom and the toxicity of *Pelagia noctiluca* (Cnidaria: Scyphozoa). A review of three decades of research in Italian laboratories and future perspectives. J. Biol. Res..

[50] Carneiro R.F.V., Nascimento N.R.F.d., Costa P.P.C., Gomes V.M., de Souza A.J.F., de Oliveira S.C.B., dos Santos Diz Filho E.B., Zara F.J., Fonteles M.C., de Oliveira Toyama D. (2011). The extract of the jellyfish *Phyllorhiza punctata* promotes neurotoxic effects. J. Appl. Toxicol..

[51] Rastogi A., Sarkar A., Chakrabarty D. (2017). Partial purification and identification of a metalloproteinase with anticoagulant activity from *Rhizostoma pulmo* (Barrel Jellyfish). Toxicon.

[52] Cariello L., Romano G., Spagnuolo A., Zanetti L. (1988). Isolation and partial characterization of Rhizolysin, a high molecular weight protein with hemolytic activity, from the jellyfish *Rhizostoma pulmo*. Toxicon.

[53] Allavena A., Mariottini G.L., Carli A.M., Contini S., Martelli A. (1998). In vitro evaluation of the cytotoxic, hemolytic and clastogenic activities of *Rhizostoma pulmo* toxin(s). Toxicon.

[54] Li C., Yu H., Liu S., Xing R., Guo Z., Li P. (2005). Factors affecting the protease activity of venom from jellyfish *Rhopilema esculentum* Kishinouye. BioOrg. Med. Chem. Lett..

[55] Yu H., Li C., Li R., Xing R., Liu S., Li P. (2007). Factors influencing hemolytic activity of venom from the jellyfish *Rhopilema esculentum* Kishinouye. Food Chem. Toxicol..

[56] Gusmani L., Avian M., Galil B., Patriarca P., Rottini G. (1997). Biologically active polypeptides in the venom of the jellyfish *Rhopilema nomadica*. Toxicon.

[57] Yoffe B., Baruchin A.M. (2004). Mediterranean jellyfish (*Rhopilema nomadica*) sting. Burns.

[58] Badré S. (2014). Bioactive toxins from stinging jellyfish. Toxicon.

[59] Li R., Yu H., Xue W., Yue Y., Liu S., Xing R., Li P. (2014). Jellyfish venomics and venom gland transcriptomics analysis of *Stomolophus meleagris* to reveal the toxins associated with sting. J. Proteom..

[60] Chung J.J., Ratnapala L.A., Cooke I.M., Yanagihara A.A. (2001). Partial purification and characterization of a hemolysin (CAH1) from Hawaiian box jellyfish (*Carybdea alata*) venom. Toxicon.

[61] Nagai H., Takuwa K., Nakao M., Ito E., Miyake M., Noda M., Nakajima T. (2000). Novel proteinaceous toxins from the box jellyfish (sea wasp) *Carybdea rastoni*. Biochem. Biophys. Res. Commun..

[62] Rottini G., Gusmani L., Parovel E., Avian M., Patriarca P. (1995). Purification and properties of a cytolytic toxin in venom of the jellyfish *Carybdea marsupialis*. Toxicon.

[63] Sánchez-Rodríguez J., Torrens E., Segura-Puertas L. (2006). Partial purification and characterization of a novel neurotoxin and three cytolysins from box jellyfish (*Carybdea marsupialis*) nematocyst venom. Arch. Toxicol..

[64] Azuma H., Sekizaki S., Satoh A., Nakajima T. (1986). Platelet aggregation caused by *Carybdea rastonii* toxins (CrTX-I, II, and III) obtained from a jellyfish, *++stonii*. Proc. Soc. Exp. Biol. Med..

[65] Avila Soria G. (2009). Molecular Characterization of *Carukia barnesi* and *Malo kingi*, Cnidaria; Cubozoa; Carybdeidae. Ph.D. Thesis.

[66] Jouiaei M., Casewell N., Yanagihara A., Nouwens A., Cribb B., Whitehead D., Jackson T., Ali S.A., Wagstaff S., Koludarov I. (2015). Firing the sting: Chemically induced discharge of cnidae reveals novel proteins and peptides from box jellyfish (*Chironex fleckeri*) venom. Toxins.

[67] Brinkman D.L., Burnell J.N. (2009). Biochemical and molecular characterisation of cubozoan protein toxins. Toxicon.

[68] Nagai H., Takuwa-Kuroda K., Nakao M., Oshiro N., Iwanaga S., Nakajima T. (2002). A novel protein toxin from the deadly box jellyfish (sea wasp, Habu-kurage) *Chiropsalmus quadrigatus*. Biosci. Biotechnol. Biochem..

[69] Ramasamy S., Isbister G.K., Seymour J.E., Hodgson W.C. (2003). The in vitro effects of two chirodropid (*Chironex fleckeri* and *Chiropsalmus* sp.) venoms: Efficacy of box jellyfish antivenom. Toxicon.

[70] Lin X.-Y., Ishida M., Nagashima Y., Kazuo S. (1996). A polypeptide toxin in the sea anemone *Actinia equina* homologous with other sea anemone sodium channel toxins: Isolation and amino acid sequence. Toxicon.

[71] Shiomi K., Ishikawa M., Yamanaka H., Kikuchi T. (1989). Isolation and properties of four serine protease inhibitors in the sea anemone *Actinia equina*. Nippon Suisan Gakkaishi.

[72] Maček P., Lebez D. (1988). Isolation and characterization of three lethal and hemolytic toxins from the sea anemone *Actinia equina* L.. Toxicon.

[73] Anderluh G., Križaj I., Štrukelj B., Gubenšek F., Maček P., Pungerčar J. (1999). Equinatoxins, pore-forming proteins from the sea anemone *Actinia equina*, belong to a multigene family. Toxicon.

[74] Pungercar J., Anderluh G., Maček P., Franc G., Štrukelj B. (1997). Sequence analysis of the cDNA encoding the precursor of equinatoxin V, a newly discovered hemolysin from the sea anemone *Actinia equina*. Biochim. Biophys. Acta (BBA) Protein Struct. Mol. Enzymol..

[75] Honma T., Minagawa S., Nagai H., Ishida M., Nagashima Y., Shiomi K. (2005). Novel peptide toxins from acrorhagi, aggressive organs of the sea anemone *Actinia equina*. Toxicon.

[76] Bellomio A., Morante K., Barlič A., Gutiérrez-Aguirre I., Viguera A.R., Gonzàlez-Mañas J.M. (2009). Purification, cloning and characterization of fragaceatoxin C, a novel actinoporin from the sea anemone *Actinia fragacea*. Toxicon.

[77] Norton R.S., Bobek G., Ivanov J.O., Thomson M., Fiala-Beer E., Moritz R.L., Simpson R.J. (1990). Purification and characterisation of proteins with cardiac stimulatory and haemolytic activity from the anemone *Actinia tenebrosa*. Toxicon.

[78] Diochot S., Schweitz H., Béress L., Lazdunski M. (1998). Sea anemone peptides with a specific blocking activity against the fast inactivating potassium channel Kv3.4. J. Biol. Chem..

[79] Uechi G.-I., Toma H., Arakawa T., Sato Y. (2010). Molecular characterization on the genome structure of hemolysin toxin isoforms isolated from sea anemone *Actineria villosa* and *Phyllodiscus semoni*. Toxicon.

[80] Oshiro N., Kobayashi C., Iwanaga S., Nozaki M., Namikoshi M., Spring J.R., Nagai H. (2004). A new membrane-attack complex/perforin (MACPF) domain lethal toxin from the nematocyst venom of the Okinawan sea anemone *Actineria villosa*. Toxicon.

[81] Uechi G.-I., Toma H., Arakawa T., Sato Y. (2011). Characterization of a novel proteinous toxin from sea anemone *Actineria villosa*. Protein J..

[82] Talvinen K.A., Nevalainen T.J. (2002). Cloning of a novel phospholipase A2 from the cnidarian *Adamsia carciniopados*. Comp. Biochem. Physiol. Part B Biochem. Mol. Biol..

[83] Shiomi K., Qian W.-H., Lin X.-Y., Shimakura K., Nagashima Y., Ishida M. (1997). Novel polypeptide toxins with crab lethality from the sea anemone *Anemonia erythraea*. Biochim. Biophys. Acta (BBA) Gen. Subj..

[84] Hasegawa Y., Honma T., Nagai H., Ishida M., Nagashima Y., Shiomi K. (2006). Isolation and cDNA cloning of a potassium channel peptide toxin from the sea anemone *Anemonia erythraea*. Toxicon.

[85] Wunderer G., Eulitz M. (1978). Amino-acid sequence of toxin I from *Anemonia sulcata*. Eur. J. Biochem..

[86] Béress L., Béress R., Wunderer G. (1975). Isolation and characterisation of three polypeptides with neurotoxic activity from *Anemonia sulcata*. Febs Lett..

[87] Scheffler J.-J., Tsugita A., Linden G., Schweitz H., Lazdunski M. (1982). The amino acid sequence of toxin V from *Anemonia sulcata*. Biochem. Biophys. Res. Commun..

[88] Wunderer G., Bèress L., Machleidt W., Fritz H. (1976). Broad-specificity inhibitors from sea anemones. Methods in Enzymology.

[89] Schweitz H., Bruhn T., Guillemare E., Moinier D., Lancelin J.M., Béress L., Lazdunski M. (1995). Kalicludines and kaliseptine. Two different classes of sea anemone toxins for voltage sensitive K+ channels. J. Biol. Chem..

[90] Kohno Y., Satoh H., Iguchi A., Nagai H. (2009). Characterization of a new hemolytic protein toxin from the sea anemone *Anthopleura asiatica*. Fish. Sci..

[91] Norton T.R. (1981). Cardiotonic polypeptides from *Anthopleura xanthogrammica* (Brandt) and *A. elegantissima* (Brandt). Fed. Proc..

[92] Bruhn T., Schaller C., Schulze C., Sanchez-Rodriguez J., Dannmeier C., Ravens U., Heubach J.F., Eckhardt K., Schmidtmayer J., Schmidt H. (2001). Isolation and characterisation of five neurotoxic and cardiotoxic polypeptides from the sea anemone *Anthopleura elegantissima*. Toxicon.

[93] Diochot S., Baron A., Rash L.D., Deval E., Escoubas P., Scarzello S., Salinas M., Lazdunski M. (2004). A new sea anemone peptide, APETx2, inhibits ASIC3, a major acid-sensitive channel in sensory neurons. Embo J..

[94] Sunahara S., Muramoto K., Tenma K., Kamiya H. (1987). Amino acid sequence of two sea anemone toxins from *Anthopleura fuscoviridis*. Toxicon.

[95] Kelso G.J., Blumenthal K.M. (1998). Identification and characterization of novel sodium channel toxins from the sea anemone *Anthopleura xanthogrammica*. Toxicon.

[96] Minagawa S., Ishida M., Shimakura K., Nagashima Y., Shiomi K. (1997). Isolation and amino acid sequences of two kunitz-type protease inhibitors from the sea anemone *Anthopleura* aff. *xanthogrammica*. Comp. Biochem. Physiol. Part B Biochem. Mol. Biol..

[97] Wang L., Ou J., Peng L., Zhong X., Du J., Liu Y., Huang Y., Liu W., Zhang Y., Dong M. (2004). Functional expression and characterization of four novel neurotoxins from sea anemone *Anthopleura* sp.. Biochem. Biophys. Res. Commun..

[98] Malpezzi E.L.A., de Freitas J.C., Muramoto K., Kamiya H. (1993). Characterization of peptides in sea anemone venom collected by a novel procedure. Toxicon.

[99] Oliveira J.S., Zaharenko A.J., Ferreira W.A., Konno K., Shida C.u.S., Richardson M., Lùcio A.D., Beirão P.S.L., de Freitas J.C. (2006). BcIV, a new paralyzing peptide obtained from the venom of the sea anemone *Bunodosoma caissarum*. A comparison with the Na+ channel toxin BcIII. Biochim. Biophys. Acta (BBA) Proteins Proteom..

[100] Martins R.D., Alves R.S., Martins A.M.C., Barbosa P.S.F., Evangelista J.S.A.M., Evangelista J.J.F., Ximenes R.M., Toyama M.H., Toyama D.O., Souza A.J.F. (2009). Purification and characterization of the biological effects of phospholipase A2 from sea anemone *Bunodosoma caissarum*. Toxicon.

[101] Orts D.J.B., Moran Y., Cologna C.T., Peigneur S., Madio B., Praher D., Quinton L., De Pauw E., Bicudo J.E.P.W., Tytgat J. (2013). BcsTx3 is a founder of a novel sea anemone toxin family of potassium channel blocker. Febs J..

[102] Loret E., Menendez R., Mansuelle P., Sampieri F., Rochat H. (1994). Positively charged amino acid residues located similarly in sea anemone and scorpion toxins. J. Biol. Chem..

[103] Aneiros A., Garcìa I., Martìnez J.R., Harvey A.L., Anderson A.J., Marshall D.L., Engström Å., Hellman U., Karlsson E. (1993). A potassium channel toxin from the secretion of the sea anemone *Bunodosoma granulifera*. isolation, amino acid sequence and biological activity. Biochim. Biophys. Acta (BBA) Gen. Subj..

[104] Santana A.N.C., Leite A.B., França M.S.F., França L., Vale O.C., Cunha R.B., Ricart C.A.O., Sousa M.V., Carvalho K.M. (1998). Partial sequence and toxic effects of granulitoxin, a neurotoxic peptide from the sea anemone *Bunodosoma granulifera*. Braz. J. Med Biol. Res..

[105] Zaharenko A.J., Ferreira W.A., Oliveira J.S., Richardson M., Pimenta D.C., Konno K., Portaro F.C.V., de Freitas J.C. (2008). Proteomics of the neurotoxic fraction from the sea anemone *Bunodosoma cangicum* venom: Novel peptides belonging to new classes of toxins. Comp. Biochem. Physiol. Part D Genom. Proteom..

[106] Zaharenko A.J., Ferreira W.A., de Oliveira J.S., Konno K., Richardson M., Schiavon E., Wanke E., de Freitas J.C. (2008). Revisiting cangitoxin, a sea anemone peptide: Purification and characterization of cangitoxins II and III from the venom of *Bunodosoma cangicum*. Toxicon.

[107] Cariello L., De Santis A., Fiore F., Piccoli R., Spagnuolo A., Zanetti L., Parente A. (1989). Calitoxin, a neurotoxic peptide from the sea anemone *Calliactis parasitica*: Amino-acid sequence and electrophysiological properties. Biochemistry.

[108] Spagnuolo A., Zanetti L., Cariello L., Piccoli R. (1994). Isolation and characterization of two genes encoding calitoxins, neurotoxic peptides from *Calliactis parasitica* (Cnidaria). Gene.

[109] Ständker L., Béress L., Garateix A., Christ T., Ravens U., Salceda E., Soto E., John H., Forssmann W.-G., Aneiros A. (2006). A new toxin from the sea anemone *Condylactis gigantea* with effect on sodium channel inactivation. Toxicon.

[110] Shiomi K., Lin X.-Y., Nagashima Y., Ishida M. (1995). Isolation and aminoacid sequences of polypeptide toxins in the Caribbean Sea anemone *Condylactis passiflora*. Fish. Sci..

[111] Maeda M., Honma T., Shiomi K. (2010). Isolation and cDNA cloning of type 2 sodium channel peptide toxins from three species of sea anemones (*Cryptodendrum adhaesivum*, *Heterodactyla hemprichii* and *Thalassianthus aster*) belonging to the family Thalassianthidae. Comp. Biochem. Physiol. Part B Biochem. Mol. Biol..

[112] Ishida M., Yokoyama A., Shimakura K., Nagashima Y., Shiomi K. (1997). Halcurin, a polypeptide toxin from the sea anemone *Halcurias* sp., with a structural resemblance to type 1 and 2 toxins. Toxicon.

[113] Zykova T.A., Kozlovskaia E.P. (1989). Amino acid sequence of a neurotoxin from the anemone *Radianthus macrodactylus*. Bioorganicheskaia Khimiia.

[114] Zykova T.A., Kozlovskaia E.P., Eliakov G.B. (1988). Amino acid sequence of neurotoxin II from the sea anemone *Radianthus macrodactylus*. Bioorganicheskaia Khimiia.

[115] Zykova T.A., Kozlovskaia E.P. (1989). Disulfide bonds in neurotoxin-III from the sea anenome *Radianthus macrodactylus*. Bioorganicheskaia Khimiia.

[116] Zykova T.A., Kozlovskaia E.P., Eliakov G.B. (1988). Amino acid sequence of neurotoxins IV and V from the sea anemone *Radianthus macrodactylus*. Bioorganicheskaia Khimiia.

[117] Shiomi K., Lin X.-Y., Nagashima Y., Ishida M. (1996). Isolation and amino acid sequence of a polypeptide toxin from the sea anemone *Radianthus crispus*. Fish. Sci..

[118] Zykova T.A., Vinokurov L.M., Markova L.F., Kozlovskaya E.P., Elyakov G.B. (1985). Amino-acid sequence of trypsin inhibitor IV from *Radianthus macrodactylus*. Bioorg. Khim..

[119] Il’ina A., Lipkin A., Barsova E., Issaeva M., Leychenko E., Guzev K., Monastyrnaya M., Lukyanov S., Kozlovskaya E. (2006). Amino acid sequence of RTX-A’s isoform actinoporin from the sea anemone, *Radianthus macrodactylus*. Toxicon.

[120] Klyshko E.V., Issaeva M.P., Monastyrnaya M.M., Il’yna A.P., Guzev K.V., Vakorina T.I., Dmitrenok P.S., Zykova T.A., Kozlovskaya E.P. (2004). Isolation, properties and partial amino acid sequence of a new actinoporin from the sea anemone *Radianthus macrodactylus*. Toxicon.

[121] Kvetkina A., Leychenko E., Chausova V., Zelepuga E., Chernysheva N., Guzev K., Pislyagin E., Yurchenko E., Menchinskaya E., Aminin D. (2020). A new multigene HCIQ subfamily from the sea anemone *Heteractis crispa* encodes Kunitz-peptides exhibiting neuroprotective activity against 6-hydroxydopamine. Sci. Rep..

[122] Kozlov S.A., Osmakov D.I., Andreev Y.A., Koshelev S.G., Gladkikh I.N., Monastyrnaya M.M., Kozlovskaya E.P., Grishin E.V. (2012). A sea anemone polypeptide toxin inhibiting the ASIC3 acid-sensitive channel. Russ. J. BioOrg. Chem..

[123] Kalina R., Gladkikh I., Dmitrenok P., Chernikov O., Koshelev S., Kvetkina A., Kozlov S., Kozlovskaya E., Monastyrnaya M. (2018). New APETx-like peptides from sea anemone *Heteractis crispa* modulate ASIC1a channels. Peptides.

[124] Gladkikh I.N., Kvetkina A.N., Kostina E.E., Kalina R.S., Grebnev B.B., Koshelev S.G., Kozlov S.A., Monastyrnaya M.M., Kozlovskaya E.P. (2018). Peptide modulators of ASIC channels of the sea anemone *Urticina* aff. *coriacea* (Cuvier, 1798) from the Sea of Okhotsk. Russ. J. Mar. Biol..

[125] Gladkikh I., Monastyrnaya M., Zelepuga E., Sintsova O., Tabakmakher V., Gnedenko O., Ivanov A., Hua K.-F., Kozlovskaya E. (2015). New Kunitz-Type HCRG polypeptides from the sea anemone *Heteractis crispa*. Marine Drugs.

[126] Monastyrnaya M., Peigneur S., Zelepuga E., Sintsova O., Gladkikh I., Leychenko E., Isaeva M., Tytgat J., Kozlovskaya E. (2016). Kunitz-type peptide HCRG21 from the sea anemone *Heteractis crispa* is a full antagonist of the TRPV1 receptor. Mar. Drugs.

[127] Andreev Y.A., Kozlov S.A., Koshelev S.G., Ivanova E.A., Monastyrnaya M.M., Kozlovskaya E.P., Grishin E.V. (2008). Analgesic compound from sea anemone *Heteractis crispa* is the first polypeptide inhibitor of vanilloid receptor 1 (TRPV1). J. Biol. Chem..

[128] Andreev Y.A., Kozlov S.A., Korolkova Y.V., Dyachenko I.A., Bondarenko D.A., Skobtsov D.I., Murashev A.N., Kotova P.D., Rogachevskaja O.A., Kabanova N.V. (2013). Polypeptide modulators of TRPV1 produce analgesia without hyperthermia. Mar. Drugs.

[129] Kozlov S.A., Andreev Y.A., Murashev A.N., Skobtsov D.I., D′yachenko I.A., Grishin E.V. (2009). New polypeptide components from the *Heteractis crispa* sea anemone with analgesic activity. Russ. J. Bioorganic Chem..

[130] Tabakmakher V., Sintsova O., Krivoshapko O., Zelepuga E., Monastyrnaya M., Kozlovskaya E.P. (2015). Analgesic effect of novel Kunitz-type polypeptides of the sea anemone *Heteractis crispa*. Dokl. Biochem. Biophys..

[131] Sintsova O.V., Monastyrnaya M.M., Pislyagin E.A., Menchinskaya E.S., Leychenko E.V., Aminin D.L., Kozlovskaya E.P. (2015). Anti-inflammatory activity of a polypeptide from the *Heteractis crispa* sea anemone. Russ. J. BioOrg. Chem..

[132] Sintsova O., Pislyagin E., Gladkikh I., Monastyrnaya M., Menchinskaya E., Leychenko E., Aminin D., Kozlovskaya E. (2017). Kunitz-type peptides of the sea anemone *Heteractis crispa*: Potential anti-inflammatory compounds. Russ. J. BioOrg. Chem..

[133] Khoo K.S., Kam W.K., Khoo H.E., Gopalakrishnakone P., Chung M.C.M. (1993). Purification and partial characterization of two cytolysins from a tropical sea anemone, *Heteractis magnifica*. Toxicon.

[134] Sintsova O., Gladkikh I., Chausova V., Monastyrnaya M., Anastyuk S., Chernikov O., Yurchenko E., Aminin D., Isaeva M., Leychenko E. (2018). Peptide fingerprinting of the sea anemone *Heteractis magnifica* mucus revealed neurotoxins, Kunitz-type proteinase inhibitors and a new β-defensin α-amylase inhibitor. J. Proteom..

[135] Wang Y., Chua K.L., Khoo H.E. (2000). A new cytolysin from the sea anemone, *Heteractis magnifica*: Isolation, cDNA cloning and functional expression1. Biochim. Biophys. Acta (BBA) Protein Struct. Mol. Enzymol..

[136] Gendeh G.S., Young L.C., de Medeiros C.L.C., Jeyaseelan K., Harvey A.L., Chung M.C.M. (1997). A new potassium channel toxin from the sea anemone *Heteractis magnifica*: Isolation, cDNA cloning, and functional expression. Biochemistry.

[137] Schweitz H., Bidard J.N., Frelin C., Pauron D., Vijverberg H.P.M., Mahasneh D.M., Lazdunski M., Vilbois F., Tsugita A. (1985). Purification, sequence, and pharmacological properties of sea anemone toxins from *Radianthus paumotensis*. A new class of sea anemone toxins acting on the sodium channel. Biochemistry.

[138] Kvetkina A.N., Leychenko E.V., Yurchenko E.A., Pislyagin E.A., Peigneur S., Tytgat Y., Isaeva M.P., Aminin D.L., Kozlovskaya E.P. (2018). A new Iq-peptide of the Kunitz Type from the *Heteractis magnifica* sea anemone exhibits neuroprotective activity in a model of Alzheimer’s disease. Russ. J. BioOrg. Chem..

[139] Sintsova O., Gladkikh I., Kalinovskii A., Zelepuga E., Monastyrnaya M., Kim N., Shevchenko L., Peigneur S., Tytgat J., Kozlovskaya E. (2019). Magnificamide, a neta-defensin-like peptide from the mucus of the sea anemone *Heteractis magnifica*, Is a strong inhibitor of mammalian alpha-amylases. Mar. Drugs.

[140] Krebs H.C., Habermehl G.G. (1987). Isolierung und strukturaufklärung eines hämolytisch aktiven peptids aus der seeanemone Metridium senile. Naturwissenschaften.

[141] Logashina Y.A., Mosharova I.V., Korolkova Y.V., Shelukhina I.V., Dyachenko I.A., Palikov V.A., Palikova Y.A., Murashev A.N., Kozlov S.A., Stensvåg K. (2017). Peptide from sea anemone *Metridium senile* affects transient receptor potential ankyrin-repeat 1 (TRPA1) function and produces analgesic effect. J. Biol. Chem..

[142] Moran Y., Weinberger H., Sullivan J.C., Reitzel A.M., Finnerty J.R., Gurevitz M. (2008). Concerted evolution of sea anemone neurotoxin genes Is revealed through analysis of the *Nematostella vectensis* genome. Mol. Biol. Evol..

[143] Sachkova M.Y., Singer S.A., Macrander J., Reitzel A.M., Peigneur S., Tytgat J., Moran Y. (2019). The birth and death of toxins with distinct functions: A case study in the sea anemone *Nematostella*. Mol. Biol. Evol..

[144] Moran Y., Praher D., Schlesinger A., Ayalon A., Tal Y., Technau U. (2013). Analysis of soluble protein contents from the nematocysts of a model sea anemone sheds light on venom evolution. Mar. Biotechnol..

[145] Il’ina A.P., Monastyrnaya M.M., Isaeva M.P., Guzev K.V., Rasskazov V.A., Kozlovskaya E.P. (2005). Primary structures of actinoporins from sea anemone *Oulactis orientalis*. Russ. J. BioOrg. Chem..

[146] Nishida S., Fujita S., Warashina A., Satake M., Tamiya N. (1985). Amino acid sequence of a sea anemone toxin from *Parasicyonis actinostoloides*. Febs Lett..

[147] Mizuno M., Nozaki M., Morine N., Suzuki N., Nishikawa K., Morgan B.P., Matsuo S. (2007). A protein toxin from the sea anemone *Phyllodiscus semoni* targets the kidney and causes a severe renal injury with predominant glomerular endothelial damage. Am. J. Pathol..

[148] Nagai H., Oshiro N., Takuwa-Kuroda K., Iwanaga S., Nozaki M., Nakajima T. (2002). Novel proteinaceous toxins from the nematocyst venom of the Okinawan sea anemone *Phyllodiscus semoni* Kwietniewski. Biochem. Biophys. Res. Commun..

[149] Rodrìguez A.A., Salceda E., Garateix A.G., Zaharenko A.J., Peigneur S., Lòpez O., Pons T., Richardson M., Dìaz M., Hernàndez Y. (2014). A novel sea anemone peptide that inhibits acid-sensing ion channels. Peptides.

[150] Jiang X.-Y., Yang W.-l., Chen H.-P., Tu H.-B., Wu W.-Y., Wei J.-W., Wang J., Liu W.-H., Xu A.-L. (2002). Cloning and characterization of an acidic cytolysin cDNA from sea anemone *Sagartia rosea*. Toxicon.

[151] Kem W.R., Parten B., Pennington M.W., Price D.A., Dunn B.M. (1989). Isolation, characterization, and amino acid sequence of a polypeptide neurotoxin occurring in the sea anemone *Stichodactyla helianthus*. Biochemistry.

[152] Tysoe C., Williams L.K., Keyzers R., Nguyen N.T., Tarling C., Wicki J., Goddard-Borger E.D., Aguda A.H., Perry S., Foster L.J. (2016). Potent human alpha-amylase inhibition by the beta-defensin-like protein Helianthamide. Acs. Cent. Sci..

[153] Delfìn J., Martìnez I., Antuch W., Morera V., Gonzàlez Y., Rodìguez R., Màquez M., Saroyàn A., Larionova N., Dìaz J. (1996). Purification, characterization and immobilization of proteinase inhibitors from *Stichodactyla helianthus*. Toxicon.

[154] Castañeda O., Sotolongo V., Amor A.M., Stðcklin R., Anderson A.J., Harvey A.L., Engstrðm Å., Wernstedt C., Karlsson E. (1995). Characterization of a potassium channel toxin from the Caribbean sea anemone *Stichodactyla helianthus*. Toxicon.

[155] Huerta V., Morera V., Guanche Y., Chinea G., Gonzàlez L.J., Betancourt L., Martìnez D., Alvarez C., Lanio M.E., Besada V. (2001). Primary structure of two cytolysin isoforms from *Stichodactyla helianthus* differing in their hemolytic activity. Toxicon.

[156] Shiomi K., Honma T., Ide M., Nagashima Y., Ishida M., Chino M. (2003). An epidermal growth factor-like toxin and two sodium channel toxins from the sea anemone *Stichodactyla gigantea*. Toxicon.

[157] Honma T., Kawahata S., Ishida M., Nagai H., Nagashima Y., Shiomi K. (2008). Novel peptide toxins from the sea anemone *Stichodactyla haddoni*. Peptides.

[158] Razpotnik A., Križaj I., Kem W.R., Maček P., Turk T. (2009). A new cytolytic protein from the sea anemone *Urticina crassicornis* that binds to cholesterol- and sphingomyelin-rich membranes. Toxicon.

[159] Logashina Y.A., Solstad R.G., Mineev K.S., Korolkova Y.V., Mosharova I.V., Dyachenko I.A., Palikov V.A., Palikova Y.A., Murashev A.N., Arseniev A.S. (2017). New disulfide-stabilized fold provides sea anemone peptide to exhibit both antimicrobial and TRPA1 potentiating properties. Toxins.

[160] Osmakov D.I., Kozlov S.A., Andreev Y.A., Koshelev S.G., Sanamyan N.P., Sanamyan K.E., Dyachenko I.A., Bondarenko D.A., Murashev A.N., Mineev K.S. (2013). Sea anemone peptide with uncommon Y-hairpin structure inhibits acid-sensing ion channel 3 (ASIC3) and reveals analgesic activity. J. Biol. Chem..

[161] Cline E.I., Wiebe L.I., Young J.D., Samuel J. (1995). Toxic effects of the novel protein UPI from the sea anemone *Urticina piscivora*. Pharmacol. Res..

[162] Murakami M., Kudo I. (2002). Phospholipase A2. J. Biochem..

[163] Romero L., Marcussi S., Marchi-Salvador D., Silva F., Fuly A., Stabeli R., Da Silva S., González J., del Monte-MartÃnez A., Soares A. (2010). Enzymatic and structural characterization of a basic phospholipase A2 from the sea anemone *Condylactis gigantea*. Biochimie.

[164] Fry B.G., Roelants K., Champagne D.E., Scheib H., Tyndall J.D.A., King G.F., Nevalainen T.J., Norman J.A., Lewis R.J., Norton R.S. (2009). The toxicogenomic multiverse: Convergent recruitment of proteins into animal venoms. Annu. Rev. Genom. Hum. Genet..

[165] Undheim E.A.B., King G.F. (2011). On the venom system of centipedes (Chilopoda), a neglected group of venomous animals. Toxicon.

[166] Fox J.W., Serrano S.M.T. (2005). Structural considerations of the snake venom metalloproteinases, key members of the M12 reprolysin family of metalloproteinases. Toxicon.

[167] Messerli S.M., Greenberg R.M. (2006). Cnidarian toxins acting on voltage-gate ion channels. Mar. Drugs.

[168] Smith J.J., Blumenthal K.M. (2007). Site-3 sea anemone toxins: Molecular probes of gating mechanisms in voltage-dependent sodium channels. Toxicon.

[169] Kalina R.S., Peigneur S., Zelepuga E.A., Dmitrenok P.S., Kvetkina A.N., Kim N.Y., Leychenko E.V., Tytgat J., Kozlovskaya E.P., Monastyrnaya M.M. (2020). New insights into the Type II toxins from the sea anemone *Heteractis crispa*. Toxins.

[170] Liao Q., Feng Y., Yang B., Lee S.M.-Y. (2019). Cnidarian peptide neurotoxins: A new source of various ion channel modulators or blockers against central nervous systems disease. Drug Discov. Today.

[171] Honma T., Shiomi K. (2006). Peptide toxins in sea anemones: Structural and functional aspects. Mar. Biotechnol..

[172] Cuypers E., Yanagihara A., Karlsson E., Tytgat J. (2006). Jellyfish and other cnidarian envenomations cause pain by affecting TRPV1 channels. Febs Lett..

[173] Levine J.D., Alessandri-Haber N. (2007). TRP channels: Targets for the relief of pain. Biochim. Biophys. Acta (BBA) Mol. Basis Dis..

[174] Fernandes E.S., Fernandes M.A., Keeble J.E. (2012). The functions of TRPA1 and TRPV1: Moving away from sensory nerves. Br. J. Pharmacol..

[175] Tysoe C., Withers S.G. (2018). Structural dissection of Helianthamide reveals the basis of Its potent inhibition of human pancreaticalpha-amylase. Biochemistry.

[176] Vogg M.C., Beccari L., Iglesias OllÃ© L., Rampon C., Vriz S., Perruchoud C., Wenger Y., Galliot B. (2019). An evolutionarily-conserved Wnt3/β-catenin/Sp5 feedback loop restricts head organizer activity in *Hydra*. Nat. Commun..

[177] Chapman J.A., Kirkness E.F., Simakov O., Hampson S.E., Mitros T., Weinmaier T., Rattei T., Balasubramanian P.G., Borman J., Busam D. (2010). The dynamic genome of *Hydra*. Nature.

[178] Ohdera A., Ames C.L., Dikow R.B., Kayal E., Chiodin M., Busby B., La S., Pirro S., Collins A.G., Medina M.N. (2019). Box, stalked, and upside-down? Draft genomes from diverse jellyfish (Cnidaria, Acraspeda) lineages: *Alatina alata* (Cubozoa), *Calvadosia cruxmelitensis* (Staurozoa), and *Cassiopea xamachana* (Scyphozoa). Gigascience.

[179] Wilding C.S., Fletcher N., Smith E.K., Prentis P., Weedall G.D., Stewart Z. (2020). The genome of the sea anemone *Actinia equina* (L.): Meiotic toolkit genes and the question of sexual reproduction. Mar. Genom..

[180] Surm J.M., Stewart Z.K., Papanicolaou A., Pavasovic A., Prentis P.J. (2019). The draft genome of *Actinia tenebrosa* reveals insights into toxin evolution. Ecol. Evol..

[181] Shinzato C., Shoguchi E., Kawashima T., Hamada M., Hisata K., Tanaka M., Fujie M., Fujiwara M., Koyanagi R., Ikuta T. (2011). Using the *Acropora digitifera* genome to understand coral responses to environmental change. Nature.

[182] Ying H., Hayward D.C., Cooke I., Wang W., Moya A., Siemering K.R., Sprungala S., Ball E.E., Forêt S., Miller D.J. (2019). The whole-genome sequence of the coral *Acropora millepora*. Genome Biol. Evol..

[183] Jeon Y., Park S.G., Lee N., Weber J.A., Kim H.-S., Hwang S.-J., Woo S., Kim H.-M., Bhak Y., Jeon S. (2019). The draft genome of an octocoral, *Dendronephthya gigantea*. Genome Biol. Evol..

[184] Baumgarten S., Simakov O., Esherick L.Y., Liew Y.J., Lehnert E.M., Michell C.T., Li Y., Hambleton E.A., Guse A., Oates M.E. (2015). The genome of *Aiptasia*, a sea anemone model for coral symbiosis. Proc. Natl. Acad. Sci. USA.

[185] Helmkampf M., Bellinger M.R., Geib S.M., Sim S.B., Takabayashi M. (2019). Draft genome of the rice coral *Montipora capitata* obtained fromlLinked-read sequencing. Genome Biol. Evol..

[186] Putnam N.H., Srivastava M., Hellsten U., Dirks B., Chapman J., Salamov A., Terry A., Shapiro H., Lindquist E., Kapitonov V.V. (2007). Sea anemone genome reveals ancestral eumetazoan gene repertoire and genomic organization. Science.

[187] Prada C., Kamel B., Budd A., Woodley C., Schmutz J., Grimwood J., Iglesias-Prieto R., Pandolfi J., Levitan D., Johnson K. (2016). Empty niches after extinctions increase population sizes of modern corals. Curr. Biol..

[188] Cunning R., Bay R.A., Gillette P., Baker A.C., Traylor-Knowles N. (2018). Comparative analysis of the *Pocillopora damicornis* genome highlights role of immune system in coral evolution. Sci. Rep..

[189] Voolstra C.R., Li Y., Liew Y.J., Baumgarten S., Zoccola D., Flot J.-F., Tambutté S., Allemand D., Aranda M. (2017). Comparative analysis of the genomes of *Stylophora pistillata* and *Acropora digitifera* provides evidence for extensive differences between species of corals. Sci. Rep..

[190] Yahalomi D., Haddas-Sasson M., Rubinstein N.D., Feldstein T., Diamant A., Huchon D. (2017). The ìmultipartite mitochondrial genome of *Enteromyxum leei* (Myxozoa): Eight fast-evolving megacircles. Mol. Biol. Evol..

[191] Chang E.S., Moran N., Nimrod D.R., Arik D., Hervé P., Dorothée H., Paulyn C. (2015). Genomic insights into the evolutionary origin of Myxozoa within Cnidaria. Proc. Natl. Acad. Sci. USA.

[192] Yang Y., Xiong J., Zhou Z., Huo F., Miao W., Ran C., Liu Y., Zhang J., Feng J., Wang M. (2014). The genome of the myxosporean *Thelohanellus kitauei* shows adaptations to nutrient acquisition within its fish host. Genome Biol. Evol..

[193] Huang C., Morlighem J.-Ã.t.R.L., Zhou H., Lima Ė.P., Gomes P.B., Cai J., Lou I., Pérez C.D., Lee S.M., Ràdis-Baptista G. (2016). The transcriptome of the zoanthid *Protopalythoa variabilis* (Cnidaria, Anthozoa) predicts a basal repertoire of toxin-like and venom-auxiliary polypeptides. Genome Biol. Evol..

[194] Ames C.L., Ryan J.F., Bely A.E., Cartwright P., Collins A.G. (2016). A new transcriptome and transcriptome profiling of adult and larval tissue in the box jellyfish *Alatina alata*: An emerging model for studying venom, vision and sex. BMC. Genom..

[195] Koch T., Grimmelikhuijzen C. (2019). Global neuropeptide annotations from the genomes and transcriptomes of cubozoa, scyphozoa, staurozoa (Cnidaria: Medusozoa), and Octocorallia (Cnidaria: Anthozoa). Front. Endocrinol..

[196] Ponce D., Brinkman D.L., Potriquet J., Mulvenna J. (2016). Tentacle transcriptome and venom proteome of the Pacific sea nettle, *Chrysaora fuscescens* (Cnidaria: Scyphozoa). Toxins.

[197] Brinkman D.L., Aziz A., Loukas A., Potriquet J., Seymour J., Mulvenna J. (2012). Venom proteome of the box jellyfish *Chironex fleckeri*. PLoS ONE.

[198] Frazão B., Campos A., Osãrio H., Thomas B., Leandro S., Teixeira A., Vasconcelos V., Antunes A. (2017). Analysis of Pelagia noctiluca proteome reveals a red fluorescent protein, a zinc metalloproteinase and a peroxiredoxin. Protein J..

[199] Rachamim T., Morgenstern D., Aharonovich D., Brekhman V., Lotan T., Sher D. (2015). The dynamically evolving nematocyst content of an anthozoan, a scyphozoan, and a hydrozoan. Mol. Biol. Evol..

[200] Jaimes-Becerra A., Chung R., Morandini A.C., Weston A.J., Padilla G., Gacesa R., Ward M., Long P.F., Marques A.C. (2017). Comparative proteomics reveals recruitment patterns of some protein families in the venoms of Cnidaria. Toxicon.

[201] Lohr K.E., Khattri R.B., Guingab-Cagmat J., Camp E.F., Merritt M.E., Garrett T.J., Patterson J.T. (2019). Metabolomic profiles differ among unique genotypes of a threatened Caribbean coral. Sci. Rep..

[202] Turk T., Kem W.R. (2009). The phylum Cnidaria and investigations of its toxins and venoms until 1990. Toxicon.

[203] Prentis P.J., Pavasovic A., Norton R.S. (2018). Sea anemones: Quiet achievers in the field of peptide toxins. Toxins.

[204] Kawabata T., Lindsay D., Kitamura M., Konishi S., Nishikawa J., Nishida S., Kamio M., Nagai H. (2013). Evaluation of the bioactivities of water-soluble extracts from twelve deep-sea jellyfish species. Fish. Sci..

[205] Moritz M.I.G., Marostica L.L., Bianco E.M., Almeida M.T.R., Carraro J.L., Cabrera G.M., Palermo J.A., Simðes C.M.O., Schenkel E.P. (2014). Polyoxygenated steroids from the octocoral *Leptogorgia punicea* and in vitro evaluation of their cytotoxic activity. Mar. Drugs.

[206] Ayed Y. (2016). Evaluation of anti-proliferative and anti-inflammatory activities of *Pelagia noctiluca* venom in Lipopolysaccharide/Interferon-g stimulated RAW264.7 macrophages. Biomed. Pharmacother..

[207] Silva T., de Andrade P., Paiva-Martins F., Valentäo P., Pereira D. (2017). In vitro anti-Inflammatory and cytotoxic effects of aqueous extracts from the edible sea anemones *Anemonia sulcata* and *Actinia equina*. Int. J. Mol. Sci..

[208] Mariottini G.L., Grice I.D. (2016). Antimicrobials from Cnidarians. A new perspective for anti-infective therapy?. Mar. Drugs.

[209] Coppola D., Oliviero M., Vitale G.A., Lauritano C., D’Ambra I., Iannace S., de Pascale D. (2020). Marine collagen from alternative and sustainable sources: Extraction, processing and applications. Mar. Drugs.

[210] Lauritano C., Ferrante M.I., Rogato A. (2019). Marine natural products from microalgae: An -omics overview. Mar. Drugs.

